# During evolution from the earliest tetrapoda, newly-recruited genes are increasingly paralogues of existing genes and distribute non-randomly among the chromosomes

**DOI:** 10.1186/s12864-021-08066-3

**Published:** 2021-11-04

**Authors:** Wilfred D. Stein, Moshe B. Hoshen

**Affiliations:** 1 Silberman Institute of Life Sciences, Hebrew University, 91904 Jerusalem, Israel; 2 Bioinformatics Department, Jerusalem College of Technology, Tal Campus, Beit HaDfus 7, 95483 Jerusalem, Israel

**Keywords:** Chromosomes, Gene distribution, Newly-recruited genes, Paralogues, Phylostratigraphy, Gene ages, Gene evolution

## Abstract

**Background:**

The present availability of full genome sequences of a broad range of animal species across the whole range of evolutionary history enables one to ask questions as to the distribution of genes across the chromosomes. Do newly recruited genes, as new clades emerge, distribute at random or at non-random locations?

**Results:**

We extracted values for the ages of the human genes and for their current chromosome locations, from published sources. A quantitative analysis showed that the distribution of newly-added genes among and within the chromosomes appears to be increasingly non-random if one observes animals along the evolutionary series from the precursors of the tetrapoda through to the great apes, whereas the oldest genes are randomly distributed.

**Conclusions:**

Randomization will result from chromosome evolution, but less and less time is available for this process as evolution proceeds. Much of the bunching of recently-added genes arises from new gene formation as paralogues in gene families, near the location of genes that were recruited in the preceding phylostratum. As examples we cite the KRTAP, ZNF, OR and some minor gene families. We show that bunching can also result from the evolution of the chromosomes themselves when, as for the KRTAP genes, blocks of genes that had previously been on disparate chromosomes become linked together.

**Supplementary Information:**

The online version contains supplementary material available at 10.1186/s12864-021-08066-3.

## Background

The study of human genome evolution using orthologous genes (or orthologs) has been much furthered by Domazet-Loso and Tautz [1–3], whose pioneering phylostratigraphic approach was based on the cladistic description of evolution. A clade refers to a group of organisms containing a common ancestor and all of its descendants and has been defined as “a group of organisms that share a common evolutionary history, and are closely related, more so to members of the same group than to other organisms. These groups are recognized by sharing unique features which were not present in distant ancestors” (https://ucmp.berkeley.edu/clad/clad1.html). In the Domazet-Loso and Tautz [2] formulation, that we follow here, there are 19 successive clades that have emerged during evolution from the first living organisms to modern humans. All genes that arise in the evolutionary record between the beginning of a clade and the onset of the next clade are defined as belonging to a single phylostratum level, denoted by a number from 1 to 19. (See Table 1 at the end of this paragraph). Table 1 is based on Domazet-Loso and Tautz [2], who defined the 19 phylostrata into which they divided the genes of the organisms of the living world. (It should be emphasised that the number of phylostrata depends on the taxonomic information that is used by the researcher). In the original 2007 formulation of Domazet-Loso and Tautz [1], a phylostratum was defined as “a set of genes from an organism that coalesce to founder genes having common phylogenetic origin.” These then are the newly-appearing genes that define the relevant phylostratum level. In keeping with the original definition, we will refer to genes as being in a phylostratum, with the animals in which those genes first appeared as having a common phylogenetic origin.

**Table.1:** Definition of the 19 phylostratum levels

number	Clade beginning to onset of next	Examples of the newly-appeared organisms
1	All life up to Eukaryota	Eubacteria and bacteria
2	Eukaryota to Opisthokonta	Unicellular nucleated cells
3	Opisthokonta to Holozoa	Yeasts and molds
4	Holozoa to Metazoa	Choanoflagellates
5	Metazoa to Eumetazoa	Sponges and jellyfish
6	Eumetazoa to Bilateria	Sea anemones
7	Bilateria to Deuterostomia	Worms, limpets and octopus
8	Deuterostomia to Chordata	Sea urchins
9	Chordata to Olfactores	Lancelets
10	Olfactores to Craniata	Sea squirts
11	Craniata to Euteleostomi	Lampreys
12	Euteleostomi to Tetrapoda	Jawed fish
13	Tetrapoda to Amniota	Frogs and toads
14	Amniota to mammalia	Birds and reptiles
15	Mammalia to Eutheria	Platypus (15.1); Opossum (15.2)
16	Eutheria to Boreoeutheria	Early placental animals (elephant, armadillo)
17	Boreoeutheria to Euarchontoglires	Hoofed and pawed animals
18	Euarchontoglires to Primata	Rabbits and rodents
19	Primata	Monkeys (19.1); great apes (19.2)

The aim of a phylostratigraphic analysis of the genes of a particular organism is to ascertain for each gene X the phylostratum in which it first appeared. To do this, one finds among life’s organisms, the set of orthologs of gene X (these being those homologs of X which can be related to it by linear descent). One ranks this ortholog set into the ages of the species in which each ortholog is found. The youngest of these ages is then the age of gene X. (Arendsee and colleagues present a program for performing phylostratigraphic analyses) [4].

Applying the approach of Domazet-Loso and Tautz, we were able, for almost all of the 19,781 currently annotated protein-coding genes of the human genome, to find the consensus age of the gene (defined as the phylostratum level during which this individual gene was added to the evolving human genome) [5]. Such ages have been used to study the evolution of biological processes [6]. Here we ask: Do we find the recently-added genes to be distributed at random among the chromosomes or are such genes bunched? The two major routes by which new genes arise are, first, by the duplication of an existing gene, followed by their divergent evolution and, second, by the conversion of a non-coding sequence into a coding sequence to form a novel gene. We have found that along the vertebrates’ evolutionary path, from the precursors of the tetrapoda through to the great apes, the distribution of newly-recruited genes among the chromosomes is observed to be less and less random. We suggest a mechanism that could in part have driven this phenomenon: many newly recruited genes preferentially distribute as family members to where their older homologues had earlier arisen. As examples of this process, we consider the Zinc Finger (ZNF), the keratin-associated protein (KRTAP) and olfactory receptor (OR) gene families, and some minor families.

## Results

**Fig. 1:**
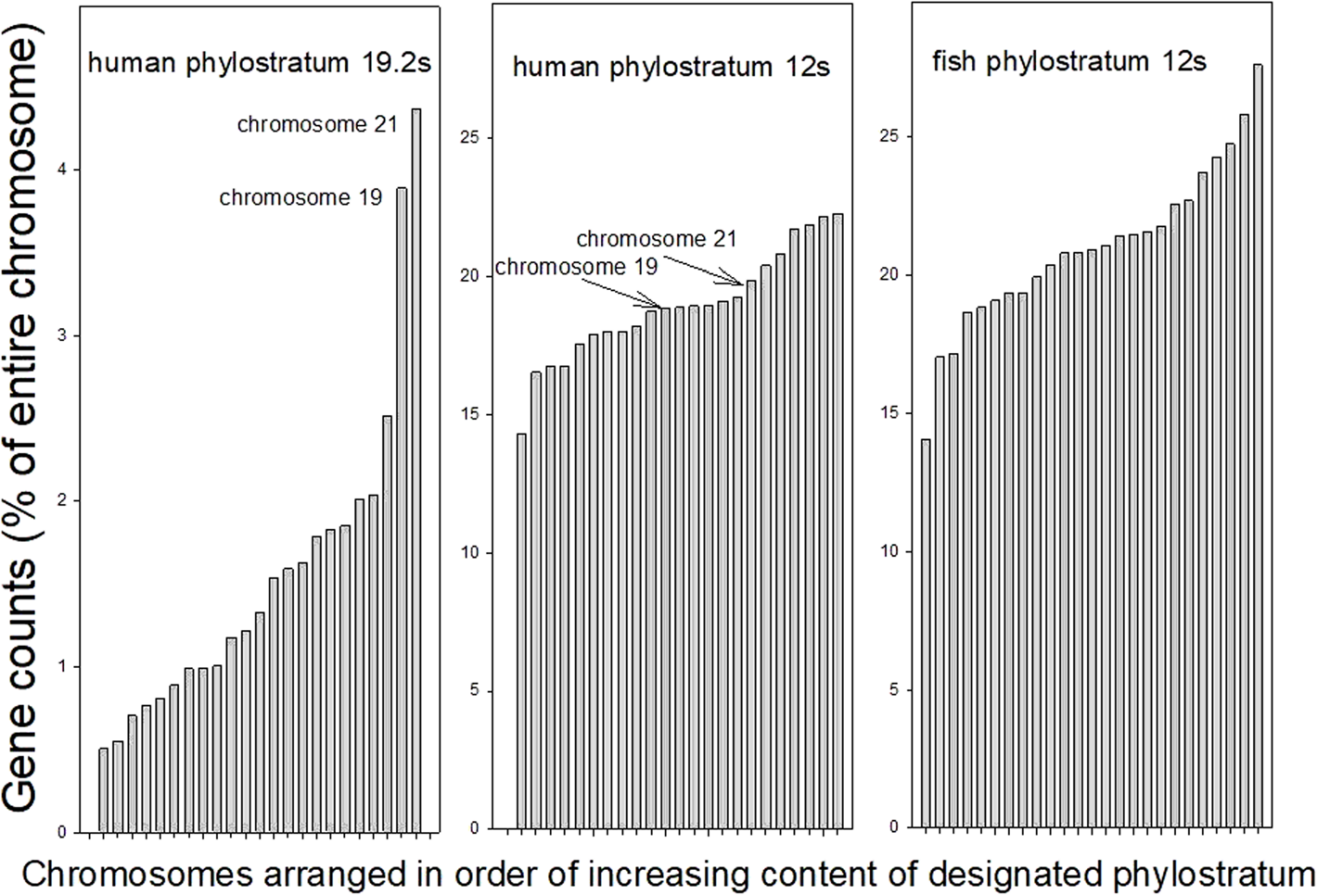
Distribution of newly recruited genes across the autosomal human chromosomes for phylostrata 19.2 (left) and 12 (middle) and for the zebrafish chromosomes for phylostratum 12 (right). The data are presented as the ratio of the content of the genes from the respective phylostratum to the gene content of the whole chromosome (as %), divided by the median of each data set and arranged in order of increasing gene content

We asked if genes that first appeared in a particular phylostratum were always distributed at random across the chromosomes of living animals, or were there perhaps contributions from particular phylostrata in which we see such genes distributed preferentially to a specific chromosome. In Fig. 1, the leftmost figure shows the % of “great ape” genes (phylostratum 19.2); the middle figure shows the “fish” genes (phylostratum 12), in both cases as they are distributed across the autosomal human chromosomes, while the rightmost figure shows the phylostratum 12 genes of the zebrafish (*Danio rero*) which, being a jawed fish, appears itself in phylostratum 12. The data that we report are, across the chromosomes of the specified animal species, the number of genes recruited in a particular phylostratum as a percentage of the total number of genes present on each chromosome, normalized by being divided by the median across that phylostratum:

We measured the spread of these values across the chromosome, at any phylostratum, using the Mean of the Absolute Deviation about the median (MAD), a standardized measure of dispersion (see Methods). It can be seen, comparing the leftmost and middle figures, that the genes that were acquired with the appearance of the great apes (with a MAD +/− SEM value of 0.38+/− 0.09, *n* = 22) are far more unevenly distributed among the human chromosomes than are the genes that were acquired with the emergence of the fish (0.05+/− 0.01, *n* = 22). The difference is significant at *P* < 0.05 (Holm-Sidak Pairwise Multiple Comparison). Comparing the middle and right figures of Fig. 1 shows that the phylostratum 12 genes are, similarly, evenly distributed also among the chromosomes of the fish itself where the MAD +/− SEM has a value of 0.08+/− 0.02, *n* = 25), the difference between these two distributions being non-significant using the same statistical test. The spread between the 75 and 25% limits as a function of the median in each case is 0.13 for the “fish” genes in the human genome while being 0.73 for the “great ape” genes. The two chromosomes with the highest fraction of phylostratum 19.2 genes (left figure, these being chromosomes 21 and 19) are no longer the most enriched compared with the phylostratum 12 genes (middle figure, where chromosome 21 is now 7th from the top and chromosome 19, 13th). To test the possibility that these results might be influenced by some gene age estimates having arisen from widely variable ortholog predictions, we chose only those genes for which the error in the age estimate (measured as the step value, see Methods, divided by the phylostratum number) was 0.5 or less. This yielded 12, 225 genes out of the total of 19,781 listed in Supplemental Table 1 of Litman and Stein [5]. Supplementary Fig. 1S depicts the results we found using this limited selection for the genes of human phylostrata 19.2 and 12, comparable to the left-hand and central panels of Fig. 1 above. The derived MAD values from this plot are 0.42 for the phylostratum 19.2 genes and 0.094 for the phylostratum 12 genes, quite comparable to the values that we derived from the full genome data set.

**Fig. 2:**
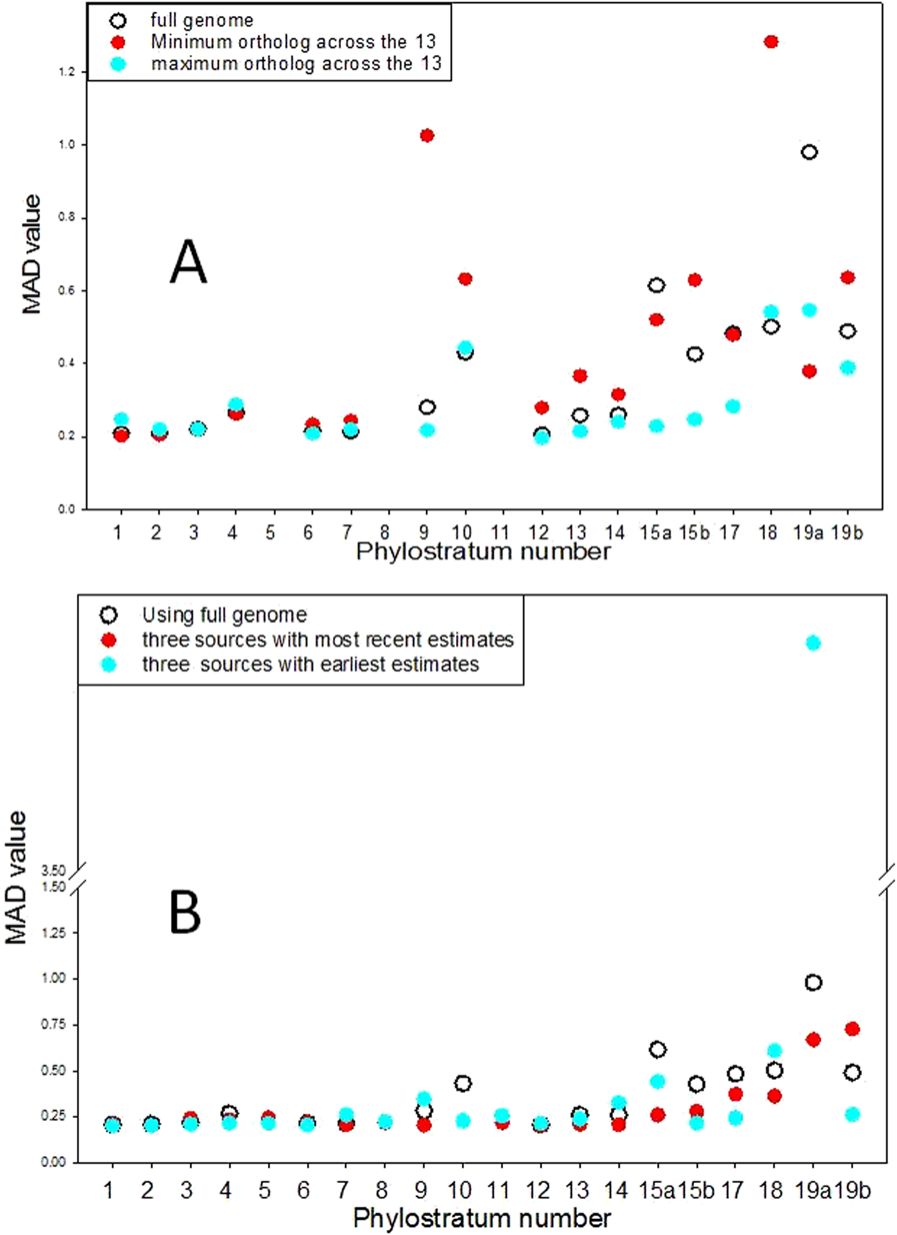
The distribution of newly recruited genes across the autosomal chromosomes of *H. sapiens*, measured as MAD values (see Methods) as a function of phylostratum number. The open circles in each figure show the data for the phylostratum numbers found as the modal values for the 13 ortholog search engines studied. The red circles in A show the data taken, for every gene, from the lowest estimate among the 13 sources while the blue circles show these for the highest estimate among the 3 sources. In B, the red circles show the data obtained as the median of those obtained for those three sources that, over the entire genome gave the lowest age estimates, while the blue circles were from the three sources that similarly gave the highest overall age estimates

Continuing this approach, we studied the random/non-random distribution of newly appearing genes, across all the chromosomes of the human genome. Figure 2 depicts these data as the open circles in both parts of the figure. To assess whether or not the choice of the consensus values for the gene ages might bias our overall finding, we plotted in part A of Fig. 2 similar plots where we chose as the gene’s age the lowest value reported for the relevant ortholog across all the 13 ortholog search engines and also the highest such value. In part B of Fig. 2 we depict plots where it is now the median value for the three ortholog search engines that have, across the human genome, the lowest overall ortholog age and also the similar data for the three search engines that have the highest overall ortholog age (see Methods for how these data were obtained).

In every case, the MAD values for the animals through the earliest tetrapoda were statistically smaller than those for the mammalia, the *P* values (Mann-Whitney Rank Sum Test) being 0.002 for the full data set,0.026 for both the minimum value chosen across the 13 search engines and for the maximum so chosen, 0.012 for the three ortholog search engines that had the lowest overall ortholog age and *P* < 0.001 for the three that had the most recent ortholog age overall.

Thus in every case depicted in the figure, the distribution of genes between the chromosomes is more random for genes that appeared early in evolution, as suggested by ortholog age estimates, than for the genes that arose in more recent epochs.

**Fig. 3:**
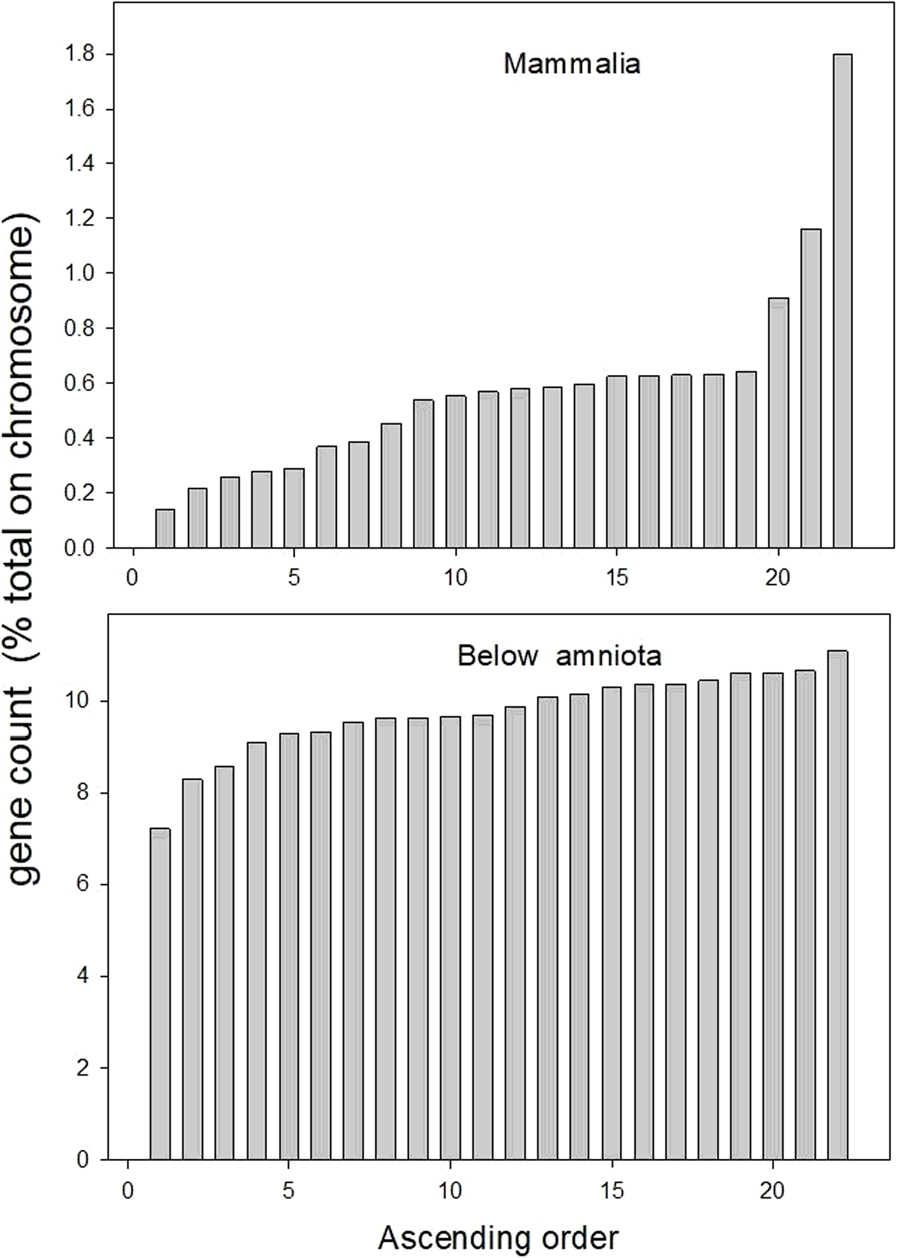
Distribution of newly recruited genes across the autosomal human chromosomes. A: All the genes assigned to phylostrata 15 through 19 by a majority of the 13 ortholog search engines. B: Those similarly assigned to phylostrata 1 through 13. The data are presented as the ratio of the total number of the genes from the respective phylostratum grouping to the gene content of the whole chromosome (as %), and arranged in order of increasing % gene content. In part A, chromosome 19 is on the extreme right, whereas in part B it is on the extreme left

To test this conclusion further, we put together in one group all those genes that were suggested to have appeared before the amniota (phylostrata 1 through 13), while in a second group, we took all the genes suggested to have appeared in the mammalia (phylostrata 15 through 19). To maximize the reliability of such gene age estimates, we chose only those genes reported upon by all 13 of our ortholog search engines and that at least 7 search engines had assigned this gene either into group 1 or group 2. For each group separately, we determined the distribution of the genes between the chromosomes, phylostratum by phylostratum, as a percentage of the total number of genes on that chromosome, and computed the random or non-random pattern of this distribution using the MAD parameter as before. Figure 3 depicts the results of this analysis, arranged as for the similar type of data of Fig. 1.

For the gene distribution depicted in Fig. 3A, the later-appearing genes, the computed MAD index of randomness is 0.490, while in Fig. 3B, the early-appearing genes, the MAD value is 0.203. These MAD values are consistent with the data of Fig. 2.

Again, to test the possibility that these results might be influenced by some gene age estimates having arisen from widely variable ortholog predictions, we chose only those genes for which the error in the estimate (see description of Fig. 1S) was 0.5 or less. Supplementary Fig. 3S depicts the results we found using this limited selection of gene. The derived MAD values from this plot are 0.51 for the genes from phylostrata 15.1 and above and 0.092 for genes from phylostratum 13 and below, quite comparable to the values that we derived from the full genome data set.

**Fig. 4:**
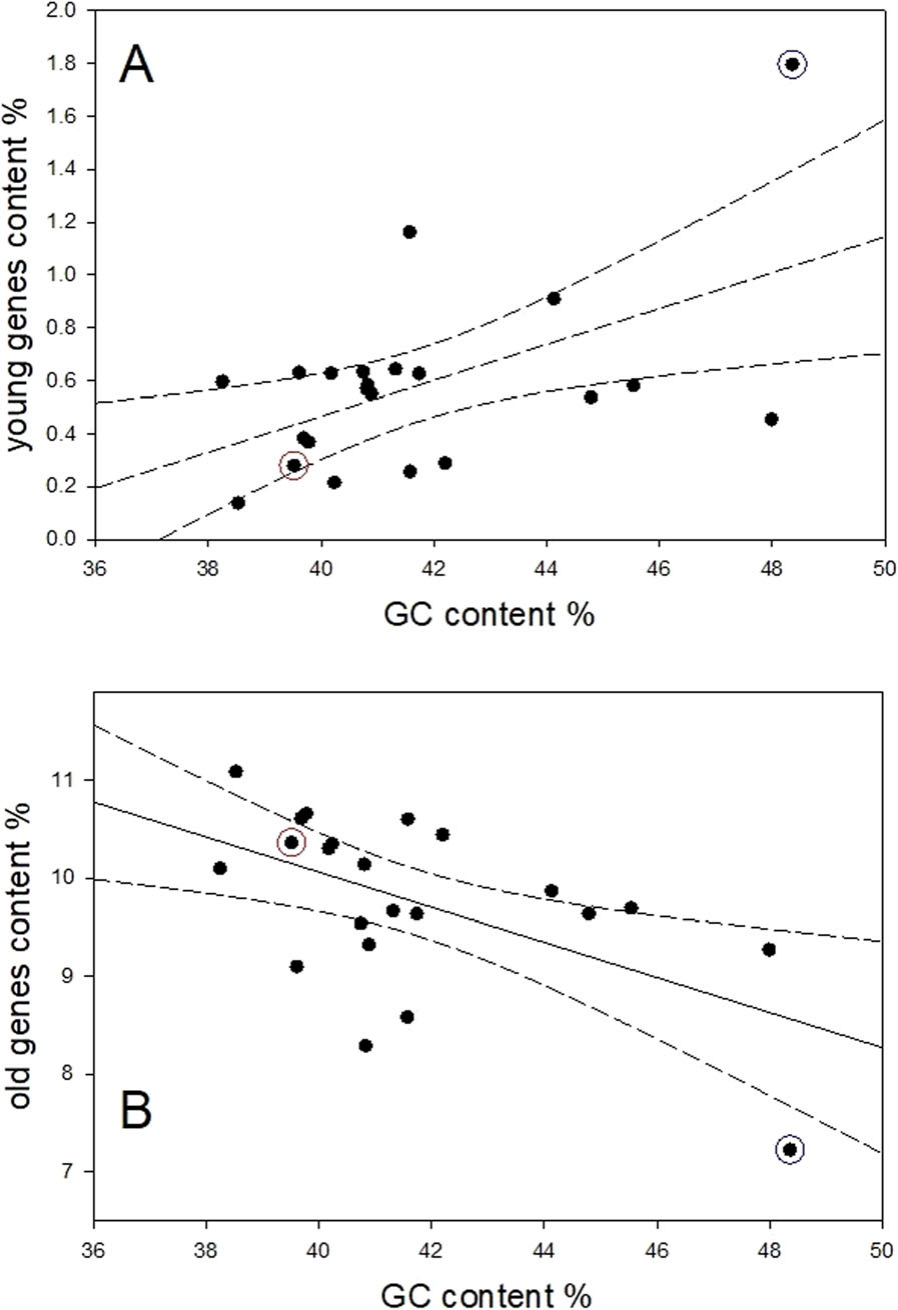
Regression of the content of (A) “young” genes (those assigned to phylostrata 15 through 19 or (B) “old” genes (those assigned to phylostrata 1 through 13) against the GC content of the chromosomes. The red-encircled points in both figures depict chromosome 5 while the blue-encircled points show chromosome 19

We wondered whether the high content of recently-incorporated genes into particular chromosomes might relate to their more open structure as reflected in their higher content of GC-rich regions [7]. To this end, we plotted, chromosome by chromosome, in Fig. 4A the content of genes with ages assigned to phylostrata 1 through 13 (“old” genes) against their content of GC genes [8], and in Fig. 4B, the content of genes assigned to phylostrata 15 through 19 (“young” genes) similarly against their GC content.

In both cases the linear regressions are statistically significant at *P* = 0.007 for the “young” genes and *P* = 0.011 for the “old” genes – but the regression lines are opposite in the sign of the slope, being positive and negative, for the “young” or “old” genes, respectively.

**Fig. 5:**
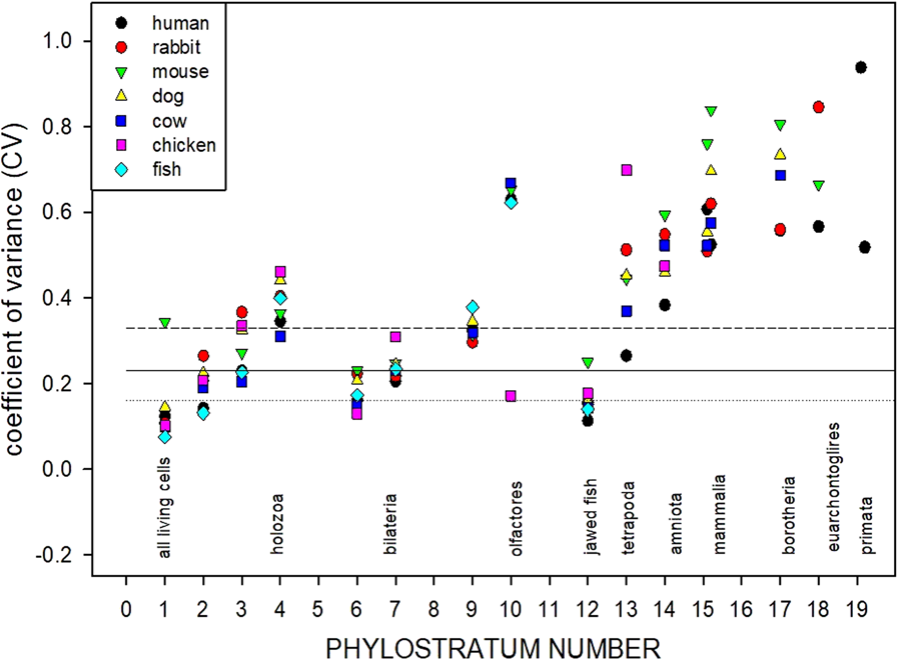
The distribution of newly recruited genes across the autosomal chromosomes, measured as MAD values – see Methods - as a function of phylostratum number, for eight animal species. The horizontal lines drawn are the median, and the 25% and the 75% limits, computed for all the data through to phylostratum 12, the euteleostomii (the jawed fish)

Extending the study using the modal age gene estimates depicted as the open circles in Fig. 2, we explored the pattern of distribution of genes across the chromosomes, phylostratum by phylostratum, across the full evolutionary trajectory for the genes of eight animal species, the genes being chosen from a range of phylostrata. Figure 5 shows the results of this analysis, plotted as the Mean Absolute Deviation about the Median (MAD – see Methods), divided by the medians, against phylostratum number for these eight animal genomes, each contributing animal species being shown by a different symbol. The raw data and computations upon which this figure is built are presented as Tables S2 through S10 in the Supplementary Materials). Note the structure of this plot: There are three data points for phylostratum 19.2, depicting the genes added in phylostratum 19.2 for the three great apes that we have analysed. Phylostratum 19.1 shows four data points, the genes that were added in phylostratum 19.1 for the three great ape species plus the data point for the macaque monkey (*Macaca mulatta*) that represents for us phylostratum 19.1. As successively earlier phylostrata are considered, further data points are added at each earlier phylostratum until, from phylostratum 12 and earlier, all eight points appear at each phylostratum number, one from each of the eight contributing species that we have used. We performed the analysis using only the autosomal genes since we were concerned that the intense evolution of, especially, the Y chromosome in the more recent phyla might affect the results. (The analysis using the whole chromosome complement is very similar to Fig. 4 (data not shown)). The horizontal lines drawn are the median, and the 25 and 75% limits, computed for all the data through to phylostratum 12, (these latter being the genes contributed by the euteleostomii, the jawed fish).

Using a mixed-effects model (see Methods), the MAD values for phylostratum 13 do not differ significantly (at *P* = 0.3711) from those for the combined phylostrata 1 through 12, but significance holds (at *P* < 0.0001) for all of the more recent phylostrata. The values for the olfactores at phylostratum 10 lie significantly above the data for the combined phylostrata 1 through 12, *P* < 0.0001). This is in spite of the extensive chromosomal rearrangements that have taken place since the time that the precursors of the olfactores appeared (See the Discussion for more on such chromosomal arrangements). The contribution of phylostratum 10 to the human genome is only 85 genes out of more than 19,000. With such a small sample number, their distribution among the 22 to 25 (in different species) autosomal chromosomes might be expected to be somewhat uneven, and variable from species to species. Indeed, the correlation coefficient between the human and macaque data for phylostratum 10 genes is not significant at *P* = 0.57, whereas the *P* value for the 19.1 phylostratum between these two species is less than 0.0001. If these outlying olfactores data are excluded from the combined phylostrata 1 through 12, the MAD values for phylostratum 13 are now significantly different from those of the combined phylostrata 1 through 12 at *P* = 0.024.

It would appear from these data that during the evolution of the vertebrates, from the precursors of the amniota (and possibly the precursors of the tetrapoda) through to the great apes, the distribution of newly recruited genes across the chromosomes appears as being increasingly non-random. An important message from this plot is this: If one looks at the MAD value for the genes that emerged with, for example, the amniotes (at phylostratum 14), their MAD number does not differ much when those “amniote” genes are seen in the chromosomes of the chicken itself, or those of the chimpanzee, macaque, or dog chromosomes. A distribution of that particular degree of scatter is found in the chromosomes of the earliest amniotes through to the great apes, although the chromosomes themselves have evolved and today vary so much between the species in size and number.

We tested whether the data could perhaps best be described by two straight lines, one horizontal and the other with a delayed slope, an ascending function of phylostratum age. An appendix in the Supplementary Methods provides the results of such an analysis. The delayed slope model was significantly superior to a single slope (*p* < 0.001).

We wondered whether the increasing patchiness seen for the newly recruited genes might in part arise from a preferred localisation to those chromosomes that had preferentially recruited genes during the immediately previous phylostratum. Table 2 records, as a matrix, the Spearman rank order correlations between the distributions of newly recruited genes across all the chromosomes, phylostratum by phylostratum. Phylostratum names, as rows and columns, are in bold. The correlation between the chromosome distribution of the genes of any phylostratum X and the distribution of those of the succeeding phylostrata, is given as the point of intersection between the rows and columns of the matrix, and is displayed as the correlation coefficient r and, directly below this, the corresponding probability P. All correlations that have *P* < 0.05 are in bold type.

**Table 2:** Spearman correlation coefficients of (autosomal) chromosome distributions between successive phylostrata in *Homo sapiens* (Bold-type values have coefficients with *P* < 0.05)

	**13**	**14**	**15.1**	**15.2**	**17**	**18**	**19.1**	**19.2**
**12**	0.0446	0.161	− 0.27	−0.211	− 0.29	−0.143	− 0.395	−0.549
***P*** **value**	0.84	0.469	0.22	0.342	0.188	0.521	0.0681	0.00823
**13**		−0.0198	−0.0593	−0.0491	0.162	0.0209	0.283	0.18
***P*** **value**		0.928	0.789	0.825	0.466	0.924	0.199	0.417
**14**			0.135	0.0288	−0.00395	−0.215	0.239	−0.246
***P*** **value**			0.544	0.896	0.984	0.332	0.28	0.266
**15.1**				**0.65**	0.248	0.0503	0.383	**0.434**
***P*** **value**				0.00104	0.262	0.821	0.077	0.0431
**15.2**					**0.659**	0.309	0.278	0.24
***P*** **value**					0.000817	0.159	0.206	0.278
**17**						**0.606**	0.23	0.233
***P*** **value**						0.00286	0.299	0.292
**18**							0.303	**0.435**
***P*** **value**							0.167	0.0425
**19.1**								**0.557**
***P*** **value**								0.00719

It will be noticed that most of the highest correlations are between successive phylostrata. These are significant except between phylostrata 18 and 19.1, where the correlation is below significance, although the correlation between phylostrata 18 and 19.2 is significant (as is that between 19.1 and 19.2). Table S11, in the Supplementary Materials shows the similar results, again for *Homo sapiens*, but now using the restricted set of more consistent data for which 3 or more ortholog databases agreed with the modal value.

We extended these between-phylostrata correlations to include a number of mammalian species. The full data set can be found as Table S12 of the Supplementary Materials.

We had noted in Fig. 1 that phylostratum 19.2 has the highest percentage of newly recruited genes on chromosome 19. We asked whether the location of newly recruited genes along chromosome 19 itself might correlate with the location of the genes recruited in the previous phylostratum. To test this, we divided chromosome 19 into twenty successive equal sections of gene counts (This gave 70 genes in each section except for the 69 genes that remained for the twentieth section). In each section, we computed the proportion of genes from any phylostratum X in chromosome 19 as a percentage of the genes of phylostratum X in the whole genome. We then performed Spearman rank order phylostratum to phylostratum correlations between the gene distributions along the twenty successive sections across chromosome 19. We asked whether the section by section content of phylostratum X genes along the chromosome correlated with the distribution of the succeeding phylostrata. The part of the data that show significant correlations is depicted in Table 3. The table records as a matrix the Spearman rank order correlations between the distribution of newly recruited genes across the twenty sections of chromosome 19, phylostratum by phylostratum. Phylostratum names appear as the rows and columns of the table. The correlation between any two phylostrata is given as the point of intersection between the rows and columns of the matrix, and is displayed as the correlation coefficient r and, below this, the corresponding probability P. All correlations that have *P* < 0.05 are in bold type, the single correlation with *P* = 0.051 is in italics.

**Table.3:** Spearman correlation coefficients of distributions along the twenty sections of chromosome 19, between successive phylostrata _ *Homo sapiens* (**Bold type** values have coefficients with *P* < 0.05, italicized value *P* = 0.051)

	15.2	17	18	19.1	19.2
**. 151**	0.31	−0.132	−0.167	− 0.232	0.0561
***P*** **value**	0.18	0.572	0.476	0.32	0.811
**15.2**		0.14	−0.0253	−0.35	− 0.184
***P*** **value**		0.551	0.911	0.127	0.429
**17**			**0.458**	0.3	0.0921
**P value**			0.0414	0.194	0.695
**18**				*0.44*	0.199
***P*** **value**				*0.051*	0.396
**19.1**					**0.698**
***P*** **value**					0.00051

Thus, significant (at *P* < 0.05) correlations exist between the distributions along the twenty sections of chromosome 19 of phylostrata 17 and 18, and 19.1 with 19.2. The correlations between non-successive phylostrata are not significant.

**Fig. 6:**
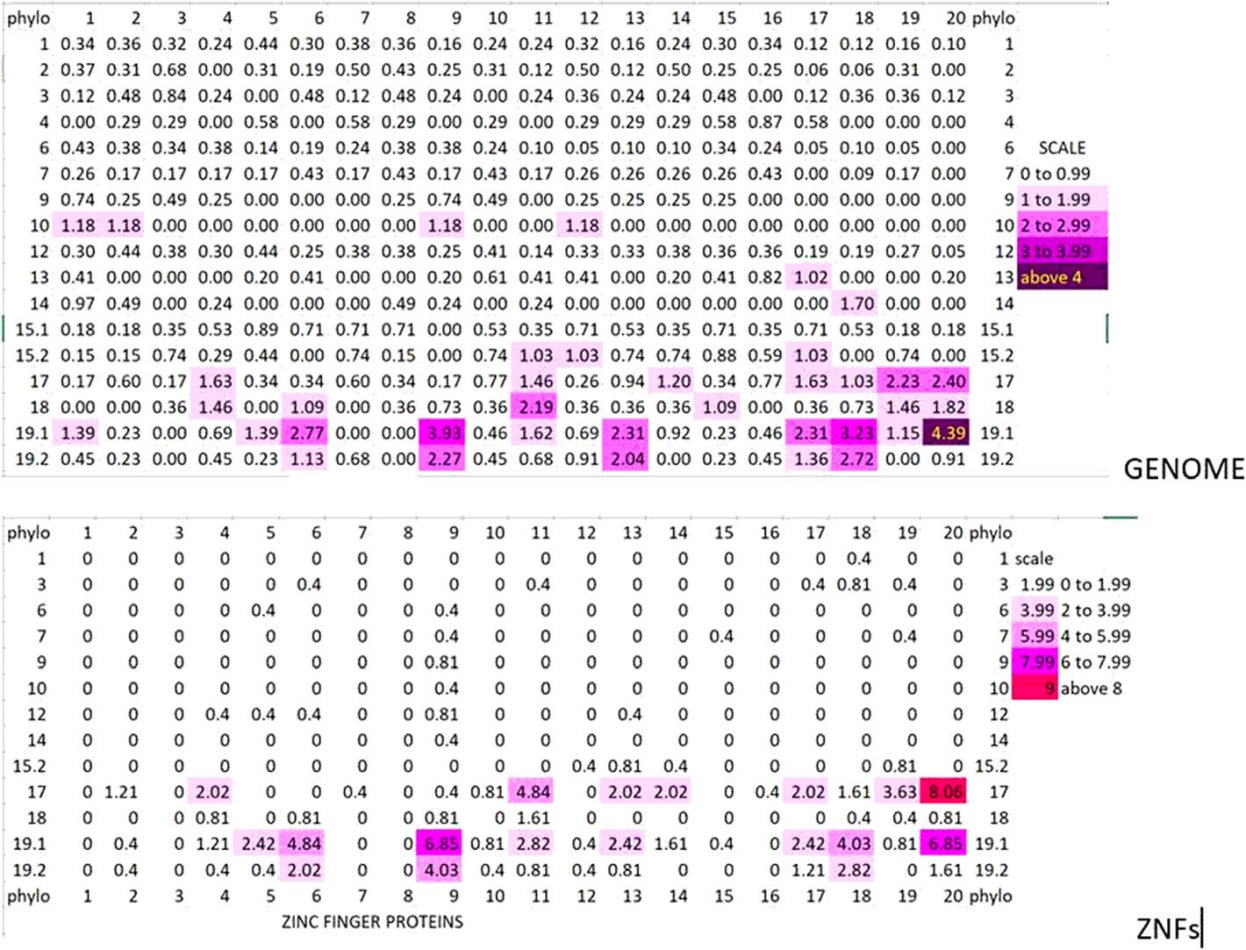
Heat maps of the distribution across the 20 sections (the columns) of human chromosome 19 of all its genes (upper figure) and of only its Zinc Finger (ZNF) genes (lower figure), the rows of the map being data for each numbered phylostratum in the 20 sections. For the full genome, the numbers are the percentage of all the genes of the phylostratum denoted, while for the ZNF data the denominator is the number of ZNF genes in the denoted phylostratum

We wanted to find out if there was a particular region of chromosome 19 at which these new gene additions, phylostratum to previous phylostratum, occurred. We used a heat map showing the gene content of the successive 20 sections of chromosome 19, comparing successive phylostrata, to provide the answer. The heat map (built as a percentage of the genes of a particular phylostratum, in a particular section, to all the genes of that phylostratum in the entire genome) is depicted in the upper part of Fig. 6:

### *The Zinc Finger (ZNF) genes of chromosome 19*

**Fig. 7:**
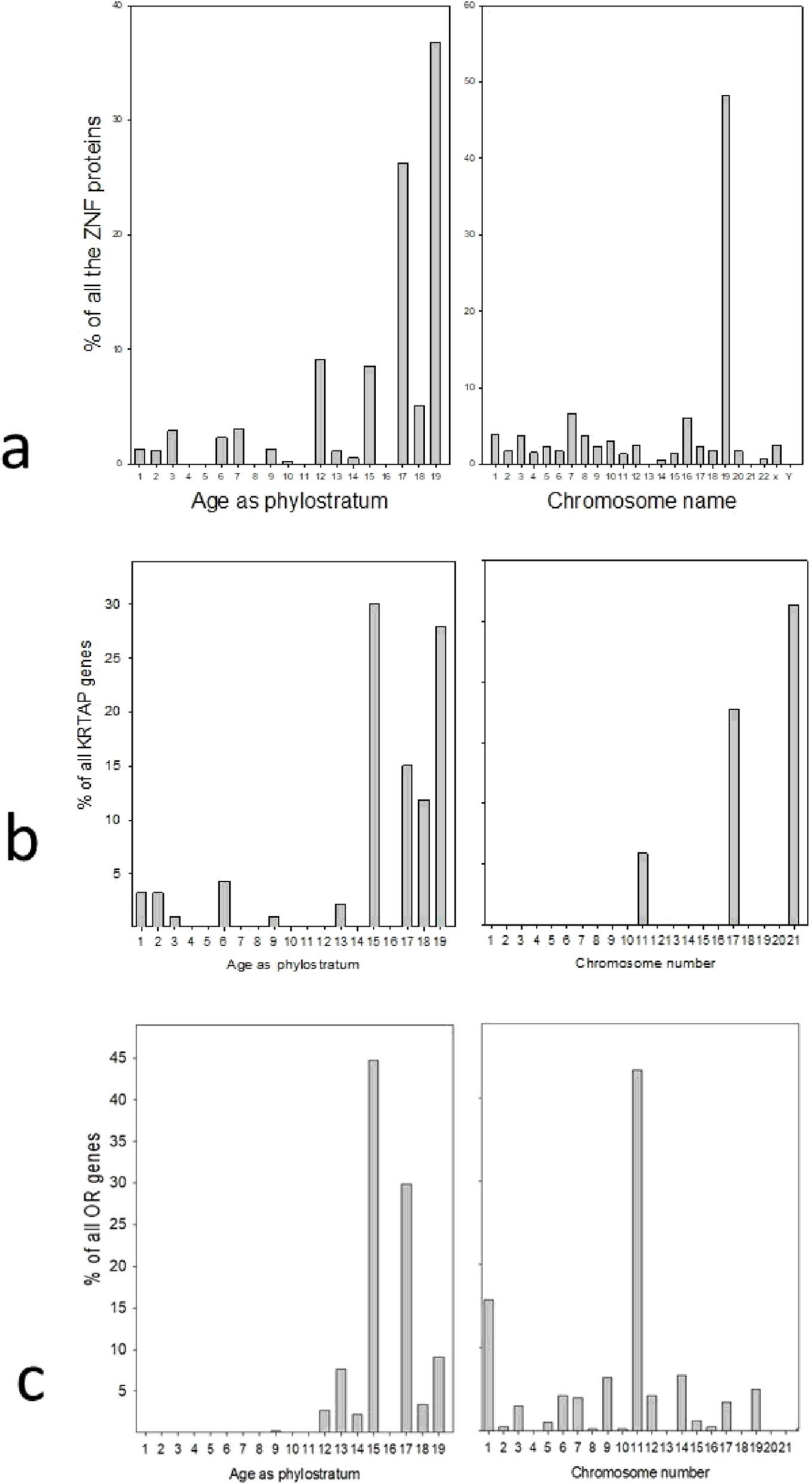
(A) The Zinc Finger (ZNF) genes of the human genome as a function of phylostratum age (left) or as their chromosome location (right); (B) similarly for the Keratin-Associated Protein (KRTAP) genes; (C) similarly again for the Olfactory Receptor (OR) genes

It would appear that the distal half of the q portion of chromosome 19 (the most distal four sections in particular) are the richest in genes that originated in recent phylostrata (17 through 19.2), those that showed the highest between- phylostrata correlations in chromosomal distributions (Table 3). Section 20 (at the distal section of the q arm of the chromosome) appears on Fig. 3 as a section with a high content of both phylostrata 17 and 19.1. This section contains a high proportion of zinc finger (ZNF) genes, these being 65% of all the genes in this section of chromosome 19. Chromosome 19 contains a high proportion of ZNF genes. Indeed, of its 1396 protein-coding genes 248 or almost 18% are ZNF genes. Figure 7A below depicts the ZNF genes of the human genome as a function of phylostratum number and chromosome location.

As can be seen, the ZNF genes are in general, recent, with most of them having been recruited in phylostrata 17 (the hoofed and pawed animals) and 19 (the primates). It is apparent, too, that chromosome 19 is to a very large extent the preferred location for these genes. We have seen that it is the most distal section of the q arm of this chromosome that is especially preferred. The distribution across chromosome 19 of the ZNF genes as a percentage of all the ZNF genes of the genome was depicted as a heat map in the lower half of Fig. 6. The map to map comparison is striking. The location of the successive cohorts of newly recruited ZNF genes appears, in many cases, to be coordinated. All of the phylostratum 19.2 genes are located at the sites where 19.1 genes had been formed, and many of the phylostratum 19.1 genes locate close to phylostratum 17 genes.

### Keratin-associated protein (KRTAP) genes

We looked for a second gene family that could be involved in between-phylostrata relations. In Fig. 1, the chromosome with the highest proportion of phylostratum 19.2 genes is chromosome 21. Now chromosome 21 is rich in genes from the KRTAP (Keratin Associated Protein) family. Figure 7B depicts the distribution of the KRTAP family genes by phylostratum age and by chromosomal location. Over 50% of these genes are located on chromosome 21 and a very high proportion of them are recent genes, largely arising with the mammals. The KRTAP genes are associated with the evolution and development of hair, a mammalian innovation [9]. The KRTAP gene family can be divided into numerous sub-families of shared evolutionary history [10], and Supplementary Fig. S2 depicts these sub-families as they are situated on chromosome 21.

### The Olfactory Receptor (OR) genes

As an additional gene family we chose the OR genes, the olfactory receptor genes. There are 406 of these in the human genome. Figure 7C shows their distribution by age and by chromosome location. Chromosome 11 is by far the richest bearer of the OR genes. Most of the OR genes were recruited with the first mammals, next highest being the hoofed and pawed animals that contributed phylostratum 17. Supplementary Fig. S3 shows the location of the OR genes along human chromosome 11. The OR genes are indeed non-randomly distributed, being present in two major complexes, with a few other solitary cases. The major complex, in the proximal region between 4.5 and 6 mB, is centered around the genes that were recruited in phylostratum 12, with the genes from phylostrata 15, 17, 18 and 19 being close by. Many of the more recent genes of chromosome 11, these being for the most part mammalian genes, were contributed by the Olfactory receptor (OR) gene family. Our prior familiarity with the ZNF, KRTAP, and OR gene families led to their being chosen as convenient samples of the class.

### Increase in randomness with evolutionary time after excluding the gene families

In addition to the three major gene families, the ZNF, KRTAP, and OR families, there are a number of minor gene families that we surmised might contribute to the non-randomness of gene distributions among the chromosomes. These minor families include the TRAV family of 45 genes, all located on chromosome 14, the TRBV family of 37 members on chromosome 7, the KIR2D and KIR3D families with 45 genes on chromosome 19, the LILR family with 33 genes on chromosome 19, and the NLRP family of 15 genes, 9 of which are located on chromosome 19. These latter genes are associated with the adaptive immune response which began to evolve with the origin of the jawed fish [11]. In addition, we took account of the PCDH gene family of 66 members, 55 of which are on chromosome 5. This list, consisting of the genes in those families that contain ten or more members, comprises 923 genes.

The genes that were incorporated into the evolving human genome as members of a gene family did so at much later epochs than those that were incorporated as an individual. Table S13 in the Supplementary Materials lists these two classes of genes together with their consensus ages while Figs. S4 and S5 in the Supplementary Materials depict these data plotted as the percentage of genes of that class (the set incorporated into families or the set incorporated as individuals) as a function of consensus age (depicted as phylostratum number in S4 or as thousands of years before present in S5). The two distributions, analysed by the Mann-Whitney Rank Sum Test (with medians of 6 and 15.2 in consensus ages) were statistically different at *p* < 0.001. It would appear that, from the earliest tetrapoda onwards, comparing the two sets, the fraction of newly-added genes that were incorporated into gene families did so significantly later than the fraction of those incorporated as individuals. At each phylostratum, the fraction of genes incorporated into a family as a proportion of all genes so incorporated increases linearly with phylostratum number (*p* = 0.010) while, in contrast, the fraction incorporated as individuals shows an insignificant decrease.

We wondered whether excluding all the genes that were incorporated into families might diminish the bunching of newly added genes that we had seen in Fig. 5 and thus asked what the MAD versus phylostratum plot might look like after excluding these major and minor gene families. The red circles in Fig. 2S in the Supplementary materials depict a plot of the uneven distribution of genes across the chromosomes of the human genome, calculated again as the appropriate MAD values, but now after excluding 923 genes, this being the total of the genes contributed by all the above-cited families. The open red triangles depict a control in which we excluded a random sample of 923 of the genes of the human genome and calculated again the appropriate MAD values.

The data points computed for the human genome after excluding these gene families differ little from the other data points until the earliest tetrapoda are reached, but then the data for the full genomes deviate increasingly upwards. This parallels the increasing fraction of genes that are in gene families which begins to deviate upwards at much the same time period (Fig. S4). The difference between the MAD values for the full human genome, for genes that were added from the earliest tetrapoda and later, is significantly different (*P* = 0.001, t-test, *N* = 7) from the data where the gene families are excluded. The difference between the MAD values for the full human genome is not significantly different (*P* = 0.301, t-test, *N* = 17) from the data where 923 genes were excluded at random. By extending the Non Linear Mixed Effect regression model to include the set with 923 randomly-excluded genes, this set could be shown to be significantly different (*P* = 0.0017, from the data set in which the gene families were excluded.

The MAD values where the gene families are excluded remain, from the earliest amniota onwards, significantly different (at *P* = 0.02, Mann-Whitney Rank Sum Test) from the data set of the MAD values through the jawed fish at phylostratum 12. This residual variance, which does not seem to arise from bunching by the previously-listed gene families, will be considered further in the discussion.

We wondered whether or not it would be useful to consider, during a particular phylostratum, the genes that were added to families already in the evolving genome separately from those genes that were added as non-related individuals. To that end we chose all the 874 genes added as the primate clade evolved, and divided these into the 417 genes that appear in the gene families listed previously (Supplementary Table S14, worksheet 2) and the remaining 457 that were added as individuals (Supplementary Table S14, worksheet 3). Fig. S6A in the Supplementary Materials shows that those genes that were recruited to the evolving primate genome as members of an existing gene family) are very unevenly distributed among the chromosomes with a MAD value of 0.52, whereas primate genes that were added as individuals were evenly distributed, with a MAD value of 0.26 (Fig. S6B).

For a comparison with published work on the emergence of new primate-specific genes, we needed to perform a search for the *H. sapiens* homologs of the KRTAP genes that had been assigned to the primate lineage in Litman and Stein [5]. To this end, using the BLAST search engine (see Methods) we identified those homologs with Expect values lower than E = 0.003 for all the KRTAP genes assigned by Litman and Stein [5] to phylostratum 19. In all cases, except for that of KRTAP20–4, we found numerous homologs (in the range 25 through 85), these being other *H. sapiens* KRTAP genes homologous to a particular KRTAP (data not shown, except for KRTAP20–4 itself and one illustrative example, KRTAP20–4, collected as sheets 1 and 3 of Table S15 of the Supplementary materials. We searched for homologs of *H. sapiens* KRTAP20–4 in all other organisms (sheet 2 of Table S15) and found matches only with KRTAP20–4 genes of the primates.

## Discussion

We used a recently published database [5] to ascertain the age of the protein-coding genes of the human genome (the age being determined by the phylostratum in which the earliest ortholog of the gene first appeared). These age estimates are probably the most reliable currently available with over 97% of the ages listed being agreed upon by three or more of the ortholog databases that were the source of the age estimates. Accepting that gene ages reported by the ortholog search engines may sometimes under-estimate an age by failing to find an occult earlier ortholog [12], we buttressed our data set by considering also the earliest age reported for a gene by any of the ortholog databases. In a variant of this approach, we considered also for any gene the median age reported by the three search engines that, across the human genome, consistently reported early ages and, finally, we considered only a subset of some two-thirds of the genes, those for which the error in the age estimate was smallest, using the “step” (defined in Methods) as a measure of this error. Our study suggested that genes that recruited early in evolution were, as a group, more randomly distributed among the chromosomes than the group of the most recently recruited genes. We decided to build on this suggestion by putting into a single group all the genes that a majority of the databases had found to be older genes (those from phylostrata 1 through 13, that is, those up to and including the amphibia). In a second group, we put all the younger genes, those that a majority of the search engines had found to be in phylostrata 15 through 19, that is, the mammalia. We reasoned that if the age of any gene in the older group had been under-estimated, it would only be shifted downwards and would thus still be in this group. The genes in the younger group are much less prone to be under-estimated since it would be most unlikely for a gene annotated, for example, in a platypus not to have its ortholog found within any of in the well-studied groups of fish, amphibians and earliest amniotes whose appearance preceded the platypus in evolutionary time. Nevertheless, if some genes that had been put into the younger group should have been placed in the group of older genes which, as Fig. 3B suggests, insert randomly among the chromosomes, their transfer out of the younger group would only raise the measure of non-randomness for the remaining young genes.

We emphasize that the approach of using orthologs to find the ages of the genes that exist in gene families does not report the single founder gene of the family but rather the earliest phylostratum at which the individual members of the family first appeared. The chromosome locations we used were drawn from the accepted source. Integrating these two sources of information, we determined the age distribution of genes across the human chromosomes. We found that genes that were added more and more recently to the evolving human genome appeared to be less and less randomly distributed in the current human chromosome complement (Fig. 5). We showed that this phenomenon (the appearance of an increasing non-random distribution) applied also to a number of other animal species that have arisen along the evolutionary path that leads to the human. At each evolutionary level, the measure of randomness of the chromosomal distribution of genes first identified in that phylostratum was little different between the genes from an animal in that phylostratum and those same genes when studied in animals from more recent phylostrata.

We searched for mechanisms that might account for this phenomenon. A major effect is probably the extensive chromosomal rearrangements that have occurred over the course of evolution [13]. In whatever pattern (bunched or scattered) newly-added genes were distributed in the earliest evolutionary epochs, chromosomal evolution would be likely to have randomized them by now. But on the evolutionary trajectory from the precursors of the tetrapoda to the primata less and less time has been available for such chromosomal reshuffling. The current pattern of gene distribution will therefore increasingly resemble the initially-formed pattern as we approach the current epoch.

There is, moreover, a second, determining process that contributes to the observed bunching of recently added genes. An increasing portion of the genes added since the tetrapoda have been paralogues, genes that were added to existing gene families (Fig. S5). As we saw in Fig. 7A, B, and C the size of three such gene families (the ZNF (zinc finger), the KRTAP (keratin-associated protein), and the OR (olfactory receptor) genes) increases with evolutionary time and their distribution among the human chromosomes is uneven. Many of the newly recruited genes will have been formed in situ in their new location by mutation after gene duplication. Often, the tandem gene duplication will have arisen through unequal crossing over, and hence. by definition localized on a particular chromosome, and thus be located within the family, bringing about bunching. Genes incorporated into gene families form an increasing proportion of newly added genes as evolution proceeds. When genes that are members of gene families are removed from our analysis, there was an almost complete elimination of the uneven distribution of genes added in the more recent phylostrata (Fig. 5, large red circles). One can eliminate the contribution of reshuffling over the ages by considering only the most recently-added genes, the primate genes. Those primate genes that were added into gene families are far more unevenly distributed among the chromosomes than are those primate genes that incorporated as individuals (Figs. S4 and S5 in the Supplementary materials). Thus, it is genes joining existing gene families that provides the basis for the phenomenon of the uneven gene distributions, coupled with the fact that insufficient time has been available for their reshuffling by chromosomal rearrangements. Even after the genes from the listed gene families are removed, there remains, however, a small, but statistically-supported, degree of non-randomness of the recently added genes. This might be due to the coming together of genes that share a common regulatory mechanism. An evolutionary advantage would result from genes becoming closely located under a single regulatory control mechanism and this would extend to genes that are not in the same family. As non-coding regulatory genes increase in number with the evolution of the higher animals, the drive to locate together genes with a common regulatory control will increase, leading to a further increase in the non-random localisation of newly recruited genes. The genes that were not added to existing gene families might perhaps be considered as true orphans in the sense that they were formed by mutational conversion of non-coding DNA sequences. Orphan genes are genes to which no homolog has been identified, but this could be because it has so distantly diverged from its homologs that we can now no longer detect it. In contrast, most of the genes that joined existing families were most probably formed by the duplication of existing genes (these being the earlier-added family members) followed by mutational divergence.

The nature of the mechanisms by which new genes are incorporated into the evolving human genome is currently a subject of vigorous research [14]. The present paper is concerned with the results of such new gene incorporation and not with the mechanism itself. Nevertheless, some of our results may perhaps be of interest to those working on these mechanisms.

Guerzoni and McLysaght [15] made a rigorous attempt to identify those human genes which had emerged de novo into the primate genome, rather than being formed by duplication of existing genes. To do this, they searched for genes that did not have orthologs in genomes below the primate level of evolution but for which they could identify an ancestral DNA sequence that was noncoding but orthologous to the gene in question. An appropriate mutation in this ancestral sequence could have led it to become coding and hence be the sought-for novel gene. By an exhaustive search, they found 35 de novo genes of which 16 were human-specific, 5 were (human + chimpanzee)-specific, and 14 (human + chimpanzee + gorilla)-specific. We could identify 29 of these genes using the GeneAnalytic program of GeneCards (see Methods). Of these 29, only 5 were protein coding. This is in marked contrast to the 441 protein-coding genes new to the Great Apes clade (hominidae) that were identified in the table of gene ages presented by Litman and Stein [5], the basis of the present paper. Of these 441, very many exist in gene families, such as the 73 OR (odor receptor) genes, the 62 ZNF (zinc finger), and the 17 KRTAP genes. One might argue, therefore, that the great majority of these new genes were not formed by the de novo process investigated by Guerzoni and McLysaght [15], as discussed above, but rather by duplication of existing sequences. A KRTAP gene, KRTAP20–4, is one of the 5 protein-coding genes in the Guerzoni and McLysaght list. When we looked for homologs of the KRTAP genes (see Table S15 in the Results section) we indeed found that KRTAP20–4 was the only member of the 17 KRTAP genes new to the hominidae that did not have numerous homologs. It had only itself and homologs in the Great Apes alone, fully confirming the finding of Guerzoni and McLysaght that this was the only de novo KRTAP gene. Incidentally, KRTAP20–4 is a very unusual member of its family. At a length of 44 amino-acids, it is the smallest of all the KRTAPs and, in the KRTAP expression database (see Methods), it is recorded as “Expressed in testis and 28 other tissues”, but not as expressed in hair, unlike so many of the KRTAP family. Another of the 5 protein-coding genes in the Guerzoni and McLysaght list is DNAH10OS listed in the Litman/Stein Table as emerging with the Great Apes while another in the Guerzoni/McLysaght list, TYMSOS, is confirmed by the orthologs list in GeneCard’s GeneAnalytics database (see Methods) as being new to the hominidae. In contrast, ARHGAP42 is listed in the Litman/Stein table as being associated with phylostratum 12, the jawed fish, a result confirmed by GeneCard’s GeneAnalytics. Finally, the fifth protein-coding gene on the Guerzoni/McLysaght list, GFOD1, is found by both Litman/Stein and GeneAnalytics to be present already in bacteria. Its presence in the Guerzoni/McLysaght list is almost certainly due to some error in gene annotation … as is so often found.

That de novo origination of novel genes is far from being the major source of novelty is supported by the study of Casola [16]. He analysed, in depth, previous reports in rodents of many putative de novo genes, taking into account also evidence from gene/gene synteny. Casola found that the majority of these previously identified de novo genes shared homology with genes from other vertebrates, or originated through gene duplication. This led him to lower by almost an order of magnitude the earlier reports of the rate per million years of de novo gene formation to a new value of 11.6 genes.

Thus, de novo gene formation, by mutational transformation and subsequent evolution of non-coding gene sequences, is only a minor contribution to the origin of novel genes. However, it is worth giving some thought to that mechanism of novel gene formation which, in contrast, occurs by mutation of duplicated genes. A high rate of gene duplication is, of course, associated with a high level of gene transcription, this in turn being facilitated by the more open form of chromosomes having regions of high GC content that lead to curvature [7]. Our findings, discussed in the Results section, show a strong positive correlation between the GC content of a chromosome and its content of younger genes, and the converse negative correlation with the content of older genes, supporting the contention of Vinogradov [7]. We found that chromosome 19 had the highest content of younger genes and the highest content of GC sequences. Indeed, in the early years of the human genome project, Grimwood and her colleagues [17] had reported on the high GC content of this chromosome and pointed out that more than 25% of the genes on this gene-dense chromosome were members of gene families (and likely to have been formed by gene duplication). In those same years, Schmutz and his colleagues [18] reported that chromosome 5, which our data had shown to have the highest content of older genes, had a lower than average content of segmental gene duplications.

In addition to the view discussed earlier that chromosome evolution can reduce bunching, we suggest that chromosome evolution can itself provide a route by which related genes can be brought together, thus reducing the random distribution of genes among the chromosomes. Studies on the synteny between the mammalian ancestral chromosomes and the human chromosomes suggest that chromosome 21 was formed by the conjunction of mammalian ancestral chromosome 4 and mammalian ancestral chromosome 16 [19, 20]. In the transition from the Mammalia to the Boreoeutheria, 9 new KRTAP genes were added to the already high KRTAP content of chromosome 21 (Fig. S2). This was accompanied by the conjunction that brought together the two blocks of genes of the KRTAP genes that had previously been located on separate ancestral mammalian chromosomes. The KRTAP genes comprise almost a quarter of the protein-coding genes on chromosome 21 and they are the only large family on this chromosome. The evolutionary drive for the conjunction that produced chromosome 21 might have been to bring, under a single mechanism of control and regulation, two blocks of KRTAP genes, which as we discuss further below, are so important for the development of the hair that reduces heat loss in warm-blooded animals. If so, this control is likely to arise from the higher-level organization of the chromosome [21, 22], since we found no evidence for a newly organized sharing of enhancers between the two halves of chromosome 21. The enhancers of the KRTAP genes on chromosome 21 act either on the chromosome 21 genes that originated in mammalian ancestral chromosome 4 only, or on those that originated in mammalian ancestral chromosome 16 only. A higher-level organization of the chromosome would allow the two blocks of KRTAP genes to be simultaneously accessible to regulation and thus ensure their coordinated expression.

We saw in Fig. 7 that gene families expand as evolution proceeds and that they are bunched on particular chromosomes. Gene families, of necessity, expand with time, since they are the result of the duplication and transformation of already existing genes, and they are bunched since such duplications are necessarily local. The emergence and expansion of new families is associated with the emergence of complexity. Speciation itself is driven by the population of newly-opened ecological niches, allowing for increased complexity.

Take, for example, the keratin-associated proteins, the KRTAPs, of Fig. 7B. These proteins are concerned with the properties of hair, wool and fur and are divided among 26 sub-families. These are closely intra-related structurally, being grouped into those encoding proteins having a high or ultra-high sulphur (contributed by cysteine) content, and those with a high content of glycine and tyrosine, see Wu, Irwin and Zhang [10]. The keratins and the keratin-associated proteins act together to form the hair fibres [23]. The different combinations determine the particular characteristics of the different types of hair, fur, wool and quills found in various animals. Studies on wool, as an important commercial product, have led to the identification of various KRTAP family members (KRTAPs 6–3 and 20–2 in particular) that convey structural properties such as fibre thickness and curliness to the hair fibres [24]. Hair is one of the defining features of the mammals. Remnants of hair fibres identified within coprolites found in Upper Permian strata (Bajdek et al. [25] provide evidence that hair was present in the most primitive mammals (see also Ji et al. [26]. The insulating property of hair is associated with the maintenance of the elevated body temperature that enabled mammals to adopt a nocturnal life style. Animals living in different environments, with the concomitant opening up of new ecological niches, have evolved appropriate selections of distinct KRTAPs (Khan et al., [27]. Writing on the evolutionary sources of the KRTAP-encoding genes, Wu, Irwin, and Zhang [10] reported that the keratin-associated proteins were confined to the mammals, while in a later paper, Wu and Irwin wrote: “As the sequence composition of the KRTAP genes have no homology with other existing genes, it is likely that the KRTAP genes originated de novo from non-genic regions.” [28]. From this one might conclude that the first KRTAP was an orphan gene, or perhaps different orphans founded the different families. The descendant genes evolved from these founders by duplication followed by mutation. Consistent with this history is the genomic localisation of the KRTAPs, their being bunched in their families, and bunched on just a few chromosomes. The ecological niche here was the initial adoption of the nocturnal life-style, while the ramification of the mammalian life-style was associated with the development of new varieties and distributions of hair fibres.

A similar argument can be made for the olfactory receptor (OR) gene family of Fig. 7C. The emergence of the tetrapoda from the sea on to dry land was associated with the initial development of receptors for air-borne signalling molecules, the odours. As evolution proceeded and the range of olfactory receptors expanded, there was a corresponding expansion of the range of odours that could be detected, with an expansion of the repertoire of food-seeking behaviors. The newly-evolved OR genes would have been formed by duplication and transformation of pre-existing orphan genes or of earlier evolved family members. Thus again, the families expanded in number but remained bunched in their localisation.

Finally, as we saw in the discussion around Fig. S2, even when the members of the gene families are removed from the gene distribution computations, there still remains some residual bunching of the more recently-appearing genes. We associate this with the evolutionary advantage of having genes bunched closely together so as to be co-ordinately controlled by appropriate non-coding genes. The appearance in the genome of non-coding genes followed in time that of the appearance of the protein-coding genes. Thus bunching associated with non-coding genes would be more prevalent in recently-appearing genes.

## Conclusion

Our conclusion from all these data is that from the precursors of the tetrapoda through to the higher apes the distribution of newly-added genes across the chromosomes is increasingly non-random. Bunching arises from the preferential location of new members of gene families to where their older family members had been established. Insufficient time has been available, in these more recent epochs, for chromosomal rearrangements to have disrupted this bunching whereas, for earlier epochs, there would have been sufficient time to bring about randomization of any non-random distributions.

## Methods

### Definition of the 19 phylostratum levels

See Table 1 in the Background section.

### The ages of the genes

To determine the ages of the genes, we have used publicly available ortholog search engines. Unfortunately, the various ortholog databases interrogated by the ortholog search engines do not have uniform criteria for identifying orthologs and, thus, for identifying the earliest ortholog and hence the age of the gene in question (see for an account of ongoing progress in this field) [29]. Indeed, the age estimate for a particular gene can vary very widely between different databases. Liebeskind, McWhite and Marcotte [30] have addressed this problem by determining, for any gene, its *consensus* age across thirteen different ortholog databases as the best estimate for its age. For each gene, one consults all of the thirteen ortholog databases in turn, asking each one for its earliest version of the gene in question (its estimate of the gene’s age). One then reports, as the gene’s consensus age, the mode, i.e. the most often reported, of the ages found by the various databases. (As just discussed, there is little uniformity in the approaches and algorithms used by these 13 ortholog databases in their search for orthologs, so their various estimates of a gene’s age can be considered to be independently contributing to the modal value). Litman and Stein [5] continued the Liebeskind et al approach by completing their coverage to include an additional 933 genes that had been annotated since the Liebeskind et al. study, so as to cover now almost all of the protein-coding genes. The median age estimates across the ortholog databases were listed for cases where no modal value could be found. Litman and Stein also refined the age estimates, using the combined data set, by assigning the genes to 1 or other of the 19 phylostrata of Fig. 1, rather than just to the 10 broader groupings that Liebeskind et al. [30] had used (where, for instance, all the mammals were grouped together). In a further subdivision, both the earliest mammals (Phylostratum 15 of Fig. 1) and the primates of phylostratum 19 were each cut into 2 subdivisions [15.1 and 15.2; 19.1 and 19.2]. Unfortunately, there are as yet insufficient sequenced genomes from phylostrata 5, 8, 11, and 16 to provide data for ortholog searches in these 4 phylostrata. Supplemental Table 1 in Litman and Stein [5] records the results of this refinement of the Liebeskind et al. study and provides the source of the data on which the present study is based. In the cases where modes could be found, Litman and Stein [5] list for each gene the number of databases that reported an earliest ortholog equal to the modal value, this being of course an indication of the consistency of the gene age estimates. For each such gene, they also determined the number of “steps” by which the age estimate of any database differed from the mode, a step being the root mean square of these differences. This gives another (here inverse) measure of consistency, and is used as such in the present paper. The values found for the consensus ages are likely to be fairly robust: for 90% of the protein-coding genes, the mode was shared by 4 or more databases while for 97%, 3 or more databases shared the same value for the earliest ortholog level. As alternative measures of gene ages we ranked the 13 ortholog search engines into an ascending order of the median found across all the ages that that search engine reported for the genes of the human genome. We found search engines which reported a set of consistently low ages through to engines reporting a consistently high set. We took for each human gene, the median age reported for the three that reported a high set and the median for the three that reported a consistently high set and took these two medians for any gene as defining a high and a low estimate for that gene’s age. Genes present in gene families are a major concern of the present paper. Finding the ortholog-based ages of the genes in such families does not identify the single founder gene of the family but rather the earliest phylostratum at which the individual members of the family first appeared.

Chromosome distributions were abstracted from the ENSEMBL database (http://asia.ensembl.org/biomart/martview/3bcc5a9ab91e10d0f37c4d06b8d640d2): We took eight representative species contributing the genes of various phylostratum levels, choosing those for which full assignments of genes to chromosomes are available. This excludes, for instance, the wallaby and rabbit genomes where many genes are still assigned to scaffolds.

#### Searching for homologs of the KRTAP genes

Searches for structural similarity amongst the KRTAPs themselves were performed using the protein BLAST (Basic Local Alignment Search Tool) program of the NCBI (https://blast.ncbi.nlm.nih.gov/Blast.cgi). We collected the top 1000 closest matches, defining these as proteins that are linked to the bait used in the BLAST search. Proteins with Expect values lower than or equal to 10^–3^ compared with the bait were defined as structurally similar and hence homologs of the bait. Proteins that were annotated as KRTAP proteins or as KRTAP-like proteins were selected from these matches and were listed as the harvest of that search.

### Gene annotations

We used the GeneAnalytics program from the GeneCards website (https://genealacart.genecards.org/Query) to translate gene annotations that were not in the form of HGNC symbols into the appropriate HGNC name.

KRTAP tissue expression data were taken from the Uniprot database (https://www.uniprot.org/).

### Statistical approaches

To assess the random/non-random distribution of the newly recruited protein-coding genes across the chromosomes, we first computed for each chromosome in turn the ratio of genes having a particular phylostratum age to all the protein-coding genes on that chromosome (expressed as a percentage). We measured the spread of these values across the chromosomes, at any phylostratum, using the Mean of the Absolute Deviation about the median (MAD), a standardized measure of dispersion. We reported the computed MAD values as their ratio to the median of these percentages. The Supplementary Materials contain a file named “GUIDE TO THE MAD COMPUTATIONS REPORTED IN TABLES S2 THROUGH S10” and another called “MAD computation engine” which is the Excel-based engine that we used to convert the matrix of phylostratum content versus chromosome number into the appropriate MAD values.

To measure the concordance between the age distributions between the successive phylostrata, we used the Spearman Rank Order Correlation routine of SigmaPlot version 11.0, from Systat Software, Inc., San Jose California USA, which returns the correlation coefficient and its *p* value for the comparisons. We used this also to explore the concordance between successive cuts along a chromosome. To explore the form of the relation between the phylostratum age (PA) and MAD, we fitted the data with the multiregression routine of Sigmaplot using a dummy variable PA = n to search for the breakpoint between two successive linear components.

To account for the repeated measure element of retesting species, we used a mixed-effects model (from the Non Linear Mixed Effect package of R).

## Supplementary Information

**Additional file 1.** .

## Data Availability

The datasets accessed and analysed during the current study are available at. 32. http://asia.ensembl.org/biomart/martview/3bcc5a9ab91e10d0f37c4d06b8d640d2 33. https://blast.ncbi.nlm.nih.gov/Blast.cgi 34. https://genealacart.genecards.org/Query 35. https://www.uniprot.org/ All data generated or analysed during this study are included in this published article and its supplementary information files.
