# Supplementary material for: During evolution from the earliest tetrapoda, newly-recruited genes are increasingly paralogues of existing genes and distribute non-randomly among the chromosomes
Source: BMC Genomics. 2021 Nov 4;22:794. doi: 10.1186/s12864-021-08066-3 (PMC8570013; doi:10.1186/s12864-021-08066-3)
Supplement: Supplementary file 1 — Additional file 1. . [file 12864_2021_8066_MOESM1_ESM.zip › SUPPLEMENTARY MATERIALS first part for integration into Supp Mat pdf.docx]

SUPPLEMENTARY MATERIALS:

During evolution from the earliest tetrapoda, newly-recruited genes are increasingly paralogues of existing genes and distribute non-randomly among the chromosomes.

SUPPLEMENTARY MATERIALS:


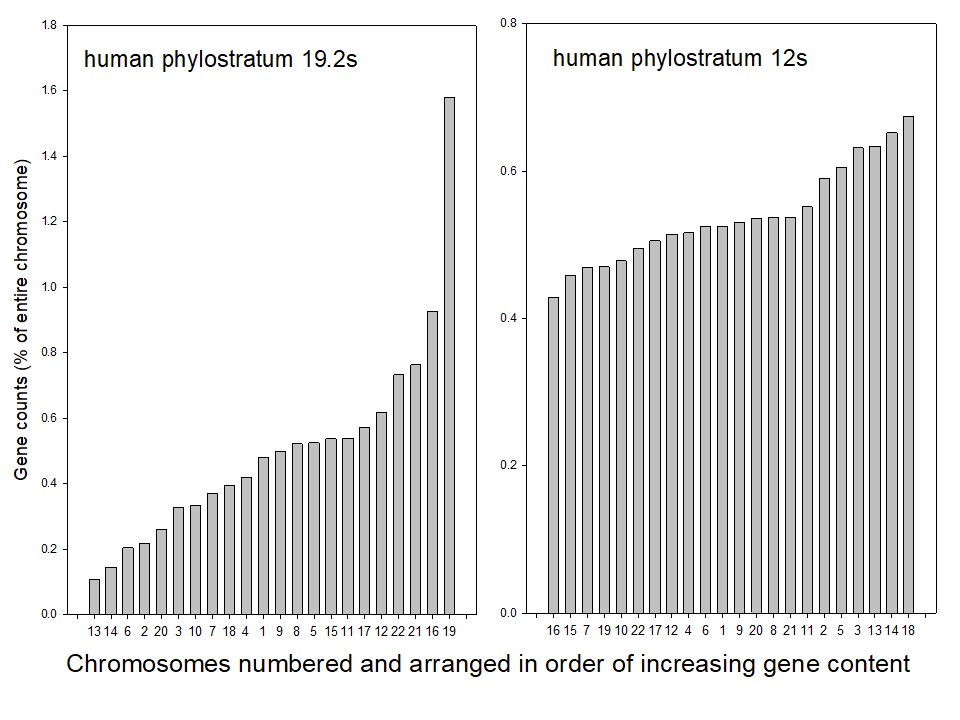


Figure 1S. Distribution of newly recruited genes across the autosomal human chromosomes for phylostrata 19.2 (left) and 12 (right), where the data analysed was a subset containing only those 12,225 genes for which the error (measured as step/phylostratum number – see text) was 0.5 or less. The data are presented as the ratio of the content of the genes from the respective phylostratum to the gene content of the whole chromosome (as %), divided by the median of each data set and arranged in order of increasing gene content. Chromosome numbers are indicated on the abscissa.


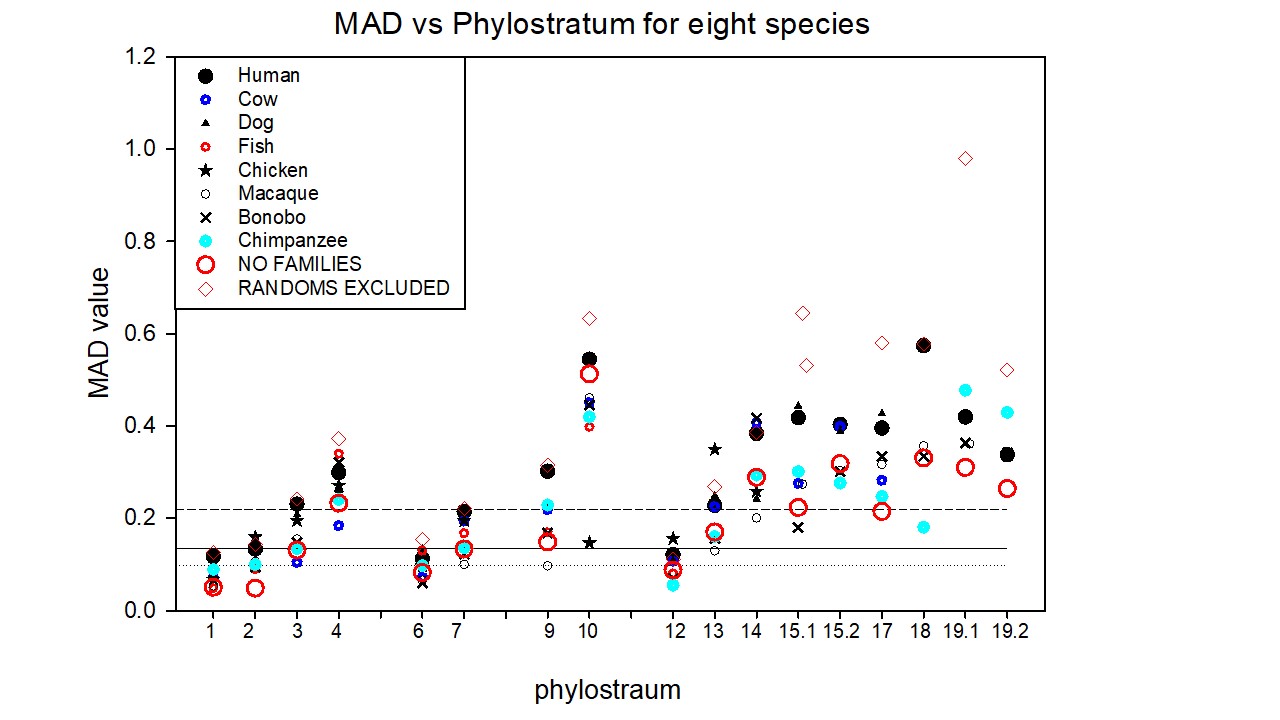


Fig. 2S (modified from Fig. 2). The distribution of newly recruited genes across the autosomal chromosomes, measured as MAD values – see Methods - as a function of phylostratum number, for eight animal species. The data points depicted as red open diamonds are for the human genome from which 923 randomly-chosen genes were excluded. The horizontal lines drawn are the median, and the 25% and the 75% limits, computed for all the data through to phylostratum 12, the euteleostomii (the jawed fish).

Figure 3S. Distribution of genes across the autosomal human chromosomes for phylostrata 15.1 and above (A, left) and 1 to 13 (B, right), where the data analysed was a subset containing only those 12,225 genes for which the error (measured as step/phylostratum number – see text) was 0.5 or less. The data are presented as the ratio of the content of the genes from the respective phylostratum to the gene content of the whole chromosome (as %), divided by the median of each data set and arranged in order of increasing gene content. Chromosome numbers are indicated on the abscissa.


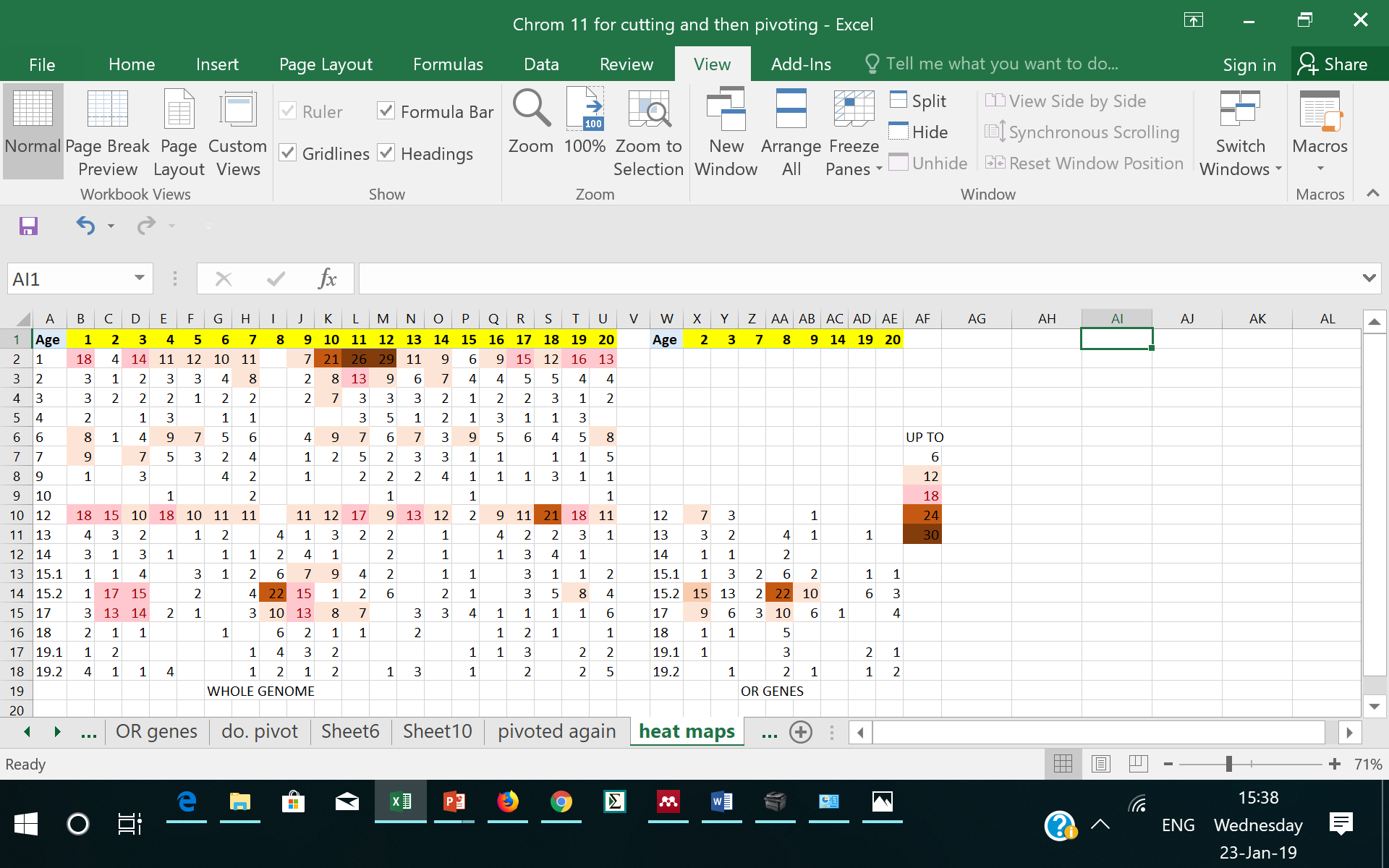


Fig. S1. Heat maps of the distribution across human chromosome 11 of all its genes (left hand figure) and of just its Olfactory receptor (OR) genes (right hand figure), the rows of the map being data for each numbered phylostratum in the 21 sections listed as columns. For both figures, the numbers shown are the absolute number of genes with the corresponding phylostratum age in that section of the chromosome. Sections that were devoid of OR genes were omitted from the right hand figure.


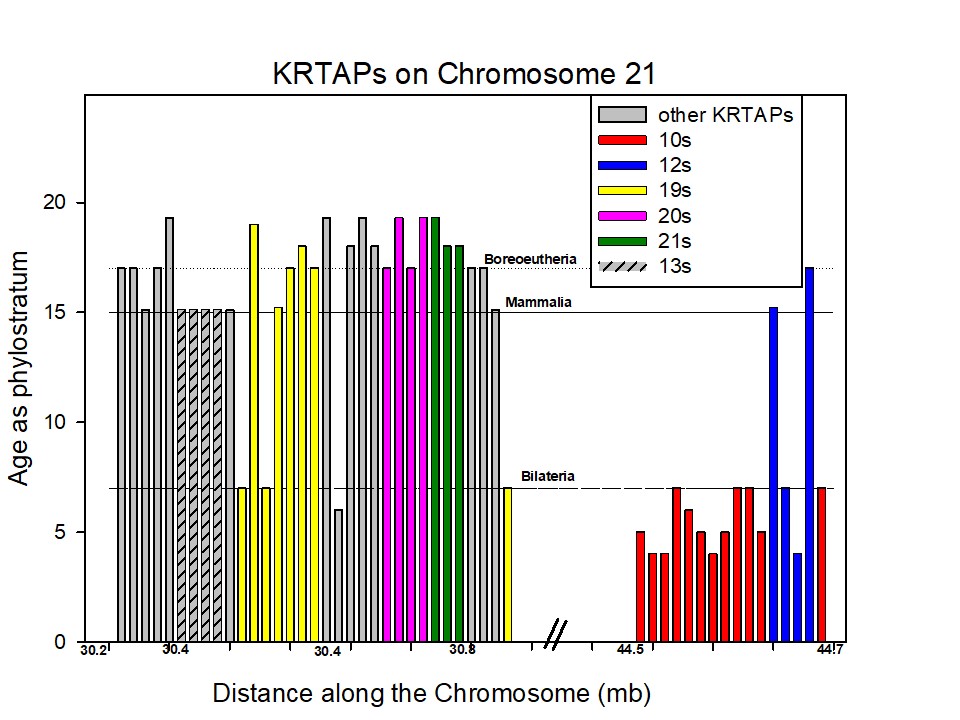


Figure S2. Location of genes of the KRTAP family along chromosome 21(in the section from 30 to 45 millibases of the 65 mB total length of this chromosome.). The sub-families of the KRTAPs are in different colours. The age (in phylostrata) of each gene is given as its height on the Y-axis.

Note that the more recent genes (from phylostrata 17 to 19), within the KRTAP subfamilies, are clustered near to those recruited during earlier phylostrata.


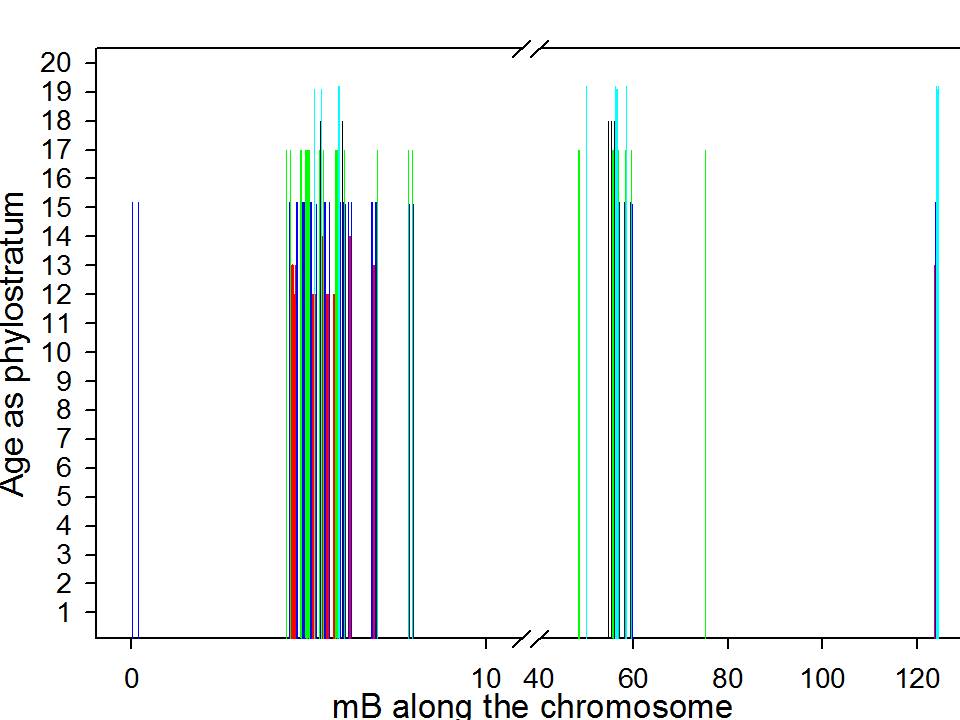


Figure S3. The location of the OR (olfactory receptor) genes along chromosome 11. The height of each bar relates to the gene age on the Y-axis. The ages are differentially coloured. The region of the break between 12 and 40 mM was devoid of OR genes.


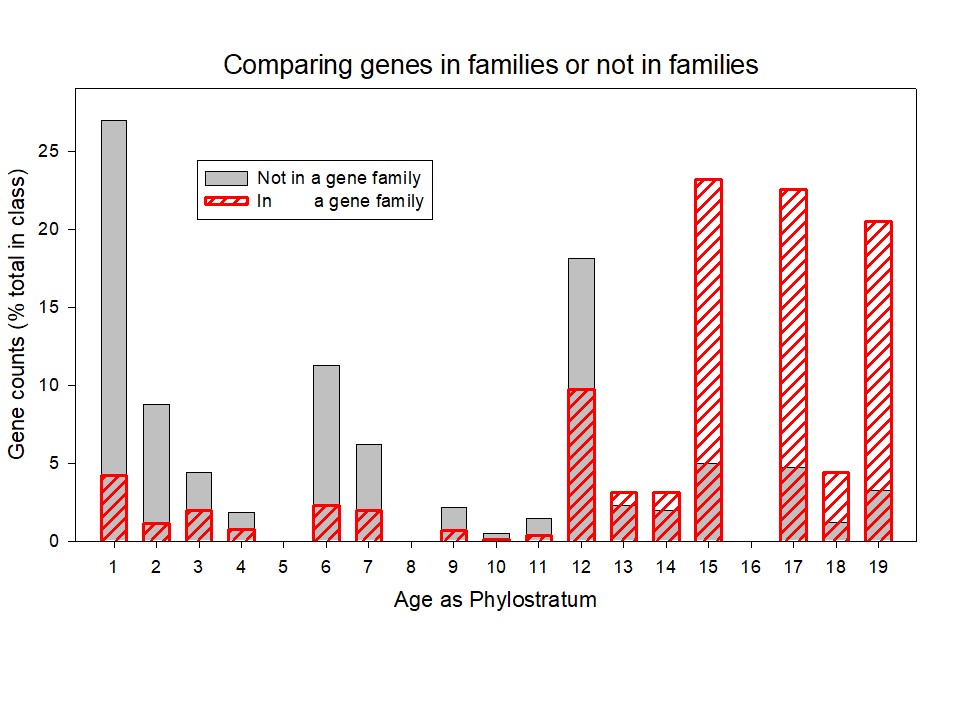


Figure S4. Comparison of the consensus age distributions of genes that were added to the human genome into families (red hatched bars) with those that were added as individuals (gray bars).


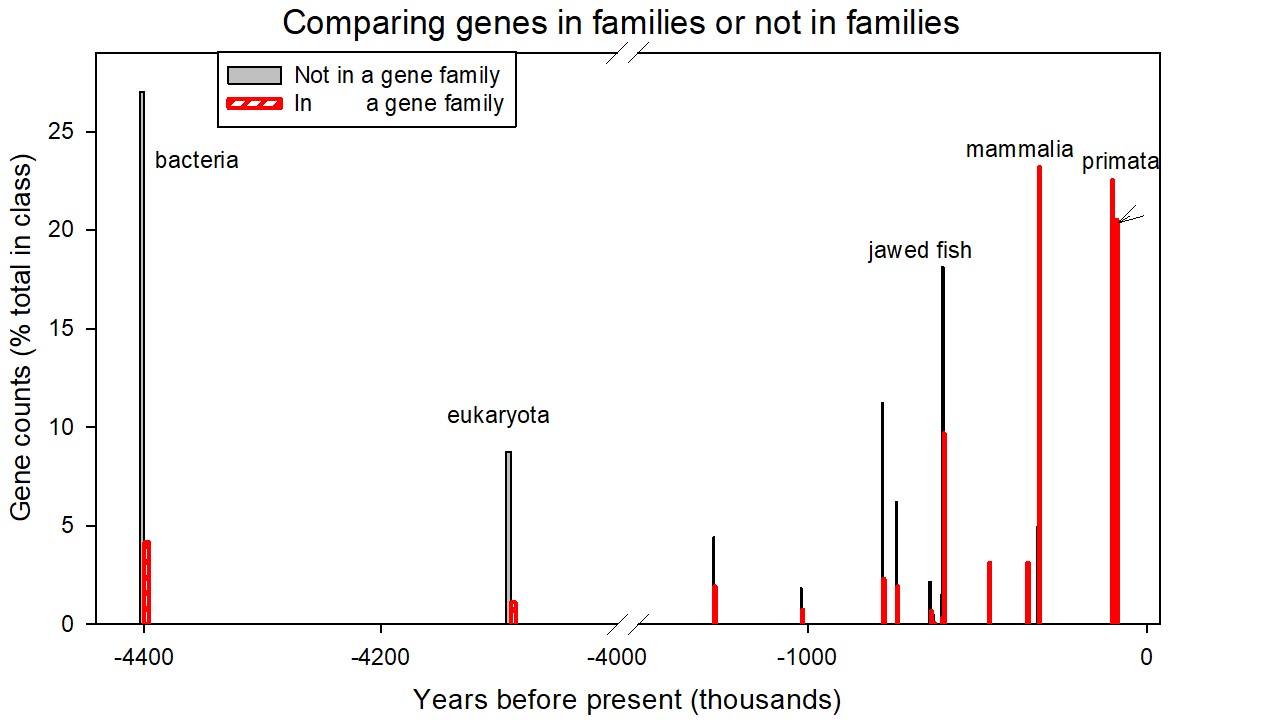


Figure S5. Comparison of the consensus age distributions of genes that were added to the human genome into families (red hatched bars) with those that were added as individuals (gray bars). The x-axis records the ages of the phylostrata in thousands of years before the present age, as taken from the Tree of Life project and recorded in the file “Times of Appearance of the Phylostrata” in these Supplementary Materials.


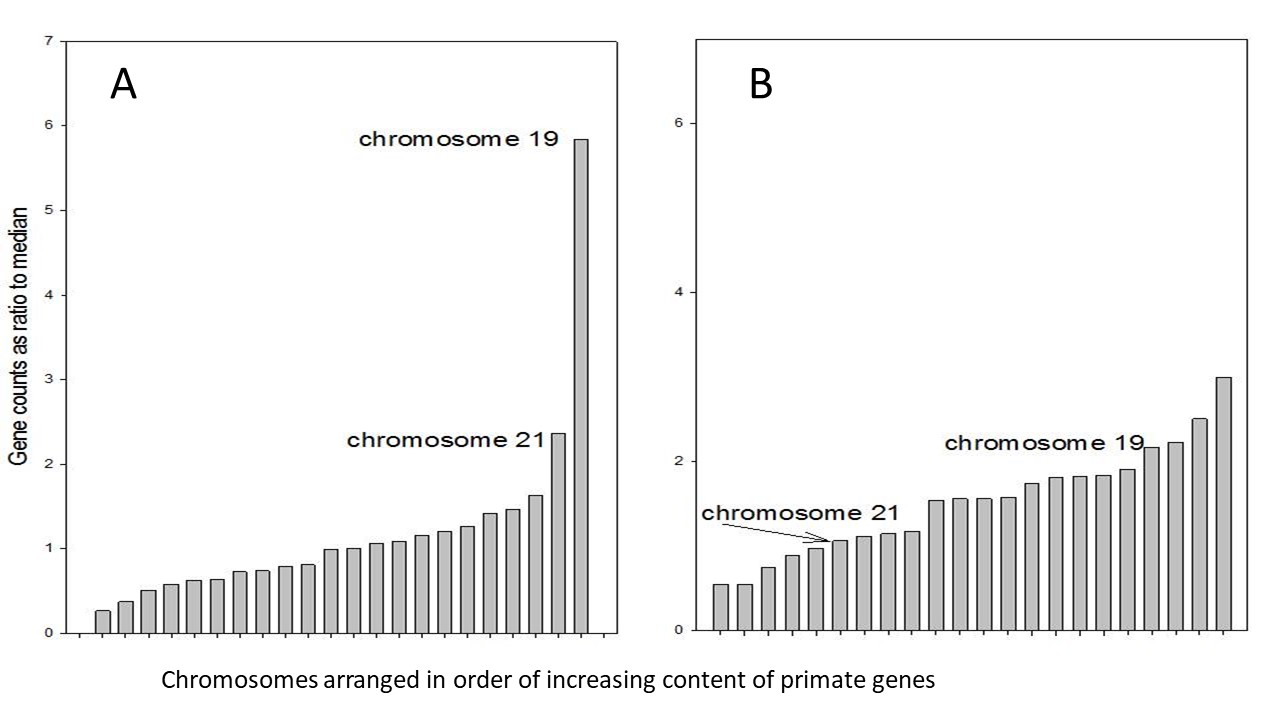


Fig S6. Chromosome distribution of the primate genes (phylostrata 19.1 and 19.2) across the human chromosome complement. The chromosomes in each section are arranged in increasing content of the primate genes. (A) The 457 genes that were in the listed (in Table S14) gene families. (B) The 417 genes that were not so listed.

Tables S2 through S10 are the Excel files labeled Table S2 through S10

Table S11

| Table S11  Spearman correlation coefficients of chromosome distributions between successive phylostrata _ Homo sapiens, using data for which 3 or more ortholog databases agreed with the modal value (**bold**-type cells have coefficients with p< 0.05) | | | | | | |
| --- | --- | --- | --- | --- | --- | --- |
|  | 15.1 | 15.2 | 17 | 18 | 19.1 | 19.2 |
| 14 | 0.0704 | 0.00522 | -0.144 | -0.25 | -0.027 | -0.302 |
| **P value** | 0.74 | 0.979 | 0.496 | 0.236 | 0.898 | 0.15 |
| 15.1 |  | **0.679** | 0.228 | -0.246 | 0.129 | 0.15 |
| **P value** |  | 0.000222 | 0.28 | 0.243 | 0.545 | 0.481 |
| 15.2 |  |  | **0.547** | 0.0191 | -0.0757 | -0.12 |
| **P value** |  |  | 0.00584 | 0.927 | 0.722 | 0.572 |
| 17 |  |  |  | **0.425** | 0.21 | 0.154 |
| **P value** |  |  |  | 0.0381 | 0.32 | 0.468 |
| 18 |  |  |  |  | 0.388 | **0.523** |
| **P value** |  |  |  |  | 0.0605 | 0.00898 |

Table S12.

| TABLE S12 | | | | | | |
| --- | --- | --- | --- | --- | --- | --- |
| SUMMARY OF ESTABLISHED SIGNIFICANT (p<0.05) BETWEEN-PHYLOSTRATA CORRELATIONS ACROSS THE CHROMOSOMES (animal species for which the indicated between phylostrata correlation was significant are listed): | | | | | | |
|  | **15.1** | **15.2** | **17** | **18** | **19.1** | **19.2** |
| **14** | mouse |  |  |  |  |  |
| **15.1** |  | Human, rabbit, cow, dog | | |  |  |
| **15.2** |  |  | Human, rabbit, cow, dog, mouse | | |  |
| **17** |  |  |  | Human, rabbit, mouse | | |
| **18** |  |  |  |  |  |  |
| **19.1** |  |  |  |  |  | Human |
| **19.2** |  |  |  |  |  |  |

Again, the significant correlations are those between successive phylostrata.

Table S13.

Table S13 is the Excel file labelled Table S13.

Table S14 is the Excel file labelled Table S14.

APPENDIX. Fitting the MAD versus phylostratum data by two straight lines

We wondered whether the data could perhaps best be described by two straight lines, one horizontal and the other an ascending function of phylostratum age PA, with a breakpoint to be determined empirically. We did not attempt to use more complex, curvilinear associations, since PA is an intrinsically non-linear variable.  To this end, we formed a linear model of (1) MAD~a+b*PA +c*PAx, where PAx represents the value of (PA-x) only for PA values greater than a breakpoint of x and is equal to 0 otherwise. The tilde ~ is the symbol for “as a function of”. The dummy parameter x varies from 0 (where there is no linear portion) to 20 (where there is no rising portion at all).

We also formed models (2) MAD ~ PA and (3) MAD ~ PAx. Both gave highly significant fits, the influences of PA and PAx on MAD being highly associated (with a variance inflation factor VIF>5). Since in the model combining PA and PAx the coefficient of PA was not significant, while that of PAx was highly significant for intermediate values of x, we were left with model 3. The R-squared value of the fit for varying values of x is plotted in Figure S9A, and the fit for the breakpoint value of x equal to 13 is shown in Figure S9B.


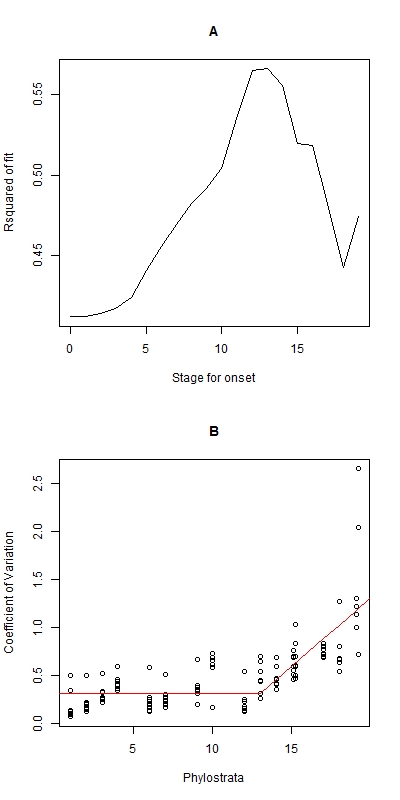


Fig. A1 Coefficient of variance MAD as a function of phylostratum ages PA (data of Fig. 2) fitted by the function MAD~ PAx, where PAx represents the value of (PA-x) only for PA values greater than a breakpoint of x and is equal to 0 otherwise. (The tilde ~ is the symbol for “as a function of”). (A) Choosing a best fit value for the inflexion point from R^2^values of plots of the defining function, computed over a range of values of the inflexion point, (B) the plot using the best-fit value of 13.
