# Supplementary material for: During evolution from the earliest tetrapoda, newly-recruited genes are increasingly paralogues of existing genes and distribute non-randomly among the chromosomes
Source: BMC Genomics. 2021 Nov 4;22:794. doi: 10.1186/s12864-021-08066-3 (PMC8570013; doi:10.1186/s12864-021-08066-3)
Supplement: Supplementary file 1 — Additional file 1. . [file 12864_2021_8066_MOESM1_ESM.zip › Table S1 Times of Appearance of the Phylostrata.docx]

| TABLE S1  Times of Appearance of the Phylostrata  (taken from the Tree of Life project^1^ | | | | |
| --- | --- | --- | --- | --- |
| Phylostratum | Clade beginning | Clade end | Median time (My) | Estimated time (My) |
| 19 | mouse | marmoset | 89 | 90 |
| 18 | horse | mouse | 94 | 96 |
| 17 | elephant | horse | 102 | 105 |
| 16 | platypus | elephant | 180 | 177 |
| 15 | lizard | platypus | 318 | 312 |
| 14 | frog | lizard | 352 | 352 |
| 13 | fish | frog | 465 | 473 |
| 12 | lamprey | cartaliginous fish | 599 | 615 |
| 11 | sea squirt | lamprey | 603 | 676 |
| 10 | lancelet | sea squirt | 637 | 684 |
| 9 | sea urchin | lancelet | 627 | 684 |
| 8 | fruitfly | sea urchin | 736 | 797 |
| 7 | sea anemone | fruitfly | 687 | 824 |
| 6 | sponge | sea anemone | 777 | 952 |
| 5 | choanoflagellate | sponge | 928 | 1023 |
| 4 | mould | choanoflagellate | 1017 | 1105 |
| 3 | rice | mould | 1275 | 1496 |
| 2 | bacteria | rice | 4090 | 4290 |
| 1 | origin of life | bacteria | 4400 | 4400 |

1: to be found at http://tolweb.org/tree/
